# Using transformers and Bi-LSTM with sentence embeddings for prediction of openness human personality trait

**DOI:** 10.7717/peerj-cs.2781

**Published:** 2025-05-22

**Authors:** Anam Naz, Hikmat Ullah Khan, Tariq Alsahfi, Mousa Alhajlah, Bader Alshemaimri, Ali Daud

**Affiliations:** 1 Department of Information Technology, University of Sargodha, Sargodha, Punjab, Pakistan; 2 Department of Information Systems and Technology, College of Computer Science and Engineering, University of Jeddah, Jeddah, Saudi Arabia; 3 Applied of Computer Science College, King Saud University, Riyadh, Saudi Arabia; 4 Software Engineering Department, College of Computing and Information Sciences, King Saud University, Riyadh, Saudi Arabia; 5 Faculty of Resilience, Rabdan Academy, Abu Dhabi, United Arab Emirates

**Keywords:** Cognitive science, Deep learning, Machine learning, Psychology, Artificial intelligence, Feature engineering

## Abstract

Understanding human personality traits is significant as it helps in decision making related to consumers’ behavior, career counselling, team building and top candidates’ selection for recruitment. Among various traits, openness is essential as it shows both diverse aspects of sensitive nature or intuitive nature. The individuals having a sensing nature tends to be more practical and prefer to focus on concrete information whereas the users having intuitive trait type is characterized by a focus on abstract ideas, creative thinking and future-oriented perspectives. In this research work, we aim to explore diverse natural language processing (NLP) based features and apply state of the art deep learning algorithms for openness trait prediction. Using standard Myers-Briggs Type Indicator (MBTI) dataset, we propose the use of the latest deep features of sentence embeddings which captures contextual semantics of the content to be used with deep learning models. For comparison, we explore textual features of Frequency-Inverse Document (TF-IDF) and parts of speech (POS) tagging with machine learning models and deep features of word2vec and global vectors for word representation (GloVe) with deep learning models. The comprehensive empirical analysis reveals that TF-IDF used with gradient boosting achieves high accuracy of 90% whereas, the deep feature of sentence embeddings when used and with deep model bidirectional long short-term memory (Bi-LSTM) achieves 90.5% accuracy. The best results have been achieved using the latest Transformer-based DistilBERT, which achieves the highest accuracy of 92% outperforming the existing studies in relevant literature.

## Introduction

Personality encompasses the unique pattern of thoughts, feelings, and behaviors that differentiate individuals from one another (Choong & Varathan, 2021). In contemporary society, social media has become a more powerful tool for the users for sharing views, opinions and comments. Such users generated content (UGC) in online social media platforms help us to have a reflective shadow on the users’ personalities (Han, Huang & Tang, 2020). In the web of digital landscape, the dynamics of social media play a significant role in shaping and modeling identities, behaviors, and perceptions that impact on users’ beliefs, ideas, emotions, and feelings. Personality, as a central focus in the lens of psychology domain, incorporates the unique set of characteristics, traits, and behaviors that define individuals’ patterns of behaving in front of media. Understanding personality is significant for acquiring deeper analysis into human behavior, decision-making processes, sentiment analysis and interpersonal dynamics (Ahmad et al., 2023). With the recent progression of artificial intelligence (AI), the significance of personality in understanding human behavior becomes even more important for examining social media content to detect and infer personality trait based on their online activities and communications (Shumanov, Cooper & Ewing, 2022). So, accurate personality detection is vital in the online ecosystem because of huge applications in diverse areas like psychology, health care, marketing, education, recommendation system (Talha et al., 2023), *etc*. The machine learning (ML) and deep learning (DL) methods have been used in the new research area of personality traits detection offering novel path for understanding and predicting human behavior (Chraibi, Chaker & Zahi, 2020). Recognizing personality type in the users enhances trust factor which is the main concern for online communication and interactions. The widely used *Personality Model* classifies a personality into four broad dimensions: Openness, Consciousness, Extroversion, and Agreeableness. Each trait offers a comprehensive spectrum of personalities that provides valuable insights into human behavior, helping to better understand how individual interact, make decisions, and respond to different environments. Such models as instrumental in applications like psychology, education and user-centric designs (Philip et al., 2019).

**Figure 1:**
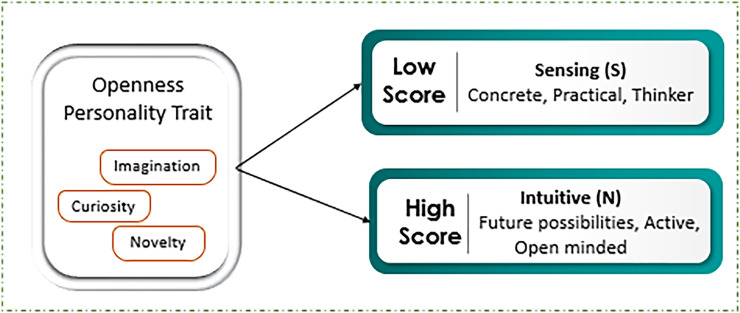
Openness mapping with scores level. The score level that indicates the behavior of individual with text whether intuitive or sensing.

Exploration of targeted trait openness is conducted by applying ML and DL approaches to investigate social media data by using framework based on Myers-Briggs Type Indicator (MBTI). By mapping MBTI data labels of N/S, representing the preferences measures of Intuitive (N) and Sensing (S) rely on sixteen personality dimensions such as Introverted, Sensing, Feeling, and Perceiving (ISFP), Extraverted, Intuitive, Feeling, and Judging (ENFJ) *etc*. Openness to experience, a central personality trait in the big five model, encompasses dimensions such as imagination, curiosity, thirst for novelty and variety, as shown in Fig. 1 (Majima & Markov, 2022). Individuals with a high level of openness tend to display strong intuitive directly, characterized by a focus on abstract thinking, future possibilities, and innovative ideas. They are often imaginative and open-minded, thriving on new experiences and unconventional approaches. Conversely, those with low level of openness preferences tend to initiate label sensing, emphasizing concrete, practical and detailed-oriented thinking. Such individuals often prefer familiarity and routine, valuing empirical evidence and sensory experiences over abstract speculation. Thus, both reflect the broader cognitive and perceptual orientations within the MBTI framework (Katare, Maurya & Kumar, 2022). Table 1 depicts the sample sentence reflecting the openness trait using NS labels depicting Intuitive *vs* Sensing nature. The sample sentences refer to behavioral patterns and cognitive processes, alongside with score that characterizes the match with the openness attributes. People with high score in Intuitive (N) self-attribute positively relate high openness characteristics such as imaginative, creative, curious, open-minded, and adventurous. The sentences in the group and related to it are characterized by such features as abstractness of thinking based on theories, rather than the existing facts. For instance, people belongings to this subject engage in introspection, and thinking through such statements as ‘I believe I’m not enemy as I have maintained moral codes that I follow,’ and ‘thoughts make me stop doing further processing on the plan’. On the other hand, for personalities with lower openness, known as Sensing (S) personalities, there are characteristics include practicality, tradition, stability, resistance, specific focus, and routine. The most connected with this group are ones expressing concern with physically verifiable aspects of reality, present-tense, everyday occurrences, deterministic and linear thinking. For example, phrases like “exploding in your face seem to me; taking it a bit too far; but hey, I can clearly see what’s happening here” are more realistic and down-to-earth. In the same vein, “As for logic I’m completely low; however as for class pace I consider myself average” is a clear example of performance orientation and self-evaluation (Jacques Junior et al., 2022).

**Table 1:** Sample sentence of NS traits.

Sentences	Trait
“I enjoy it when people do stuff | because it means. They’re giving me their precious time.”	iNtuitive—N
“ I exactly know; what thoughts make me stop to do further processing on the plan | but I can’t do anything about it.”	
“I believe I’m not enemy, as I have maintained moral codes that I follow; that I believe positively affects the people around me.”	
“Talk to him about it; be honest and direct without being confrontational.”	Sensing—S
“Blowing up in your face; seems like; a bit of overreaction; but I can definitely see what’s going on.”	
“I think; I am absolutely poor in logic; however, I am fair in class progress.”	

**Note:**

The sample of sentences that show the behavior of user personality based on dimension of Intuitive (N) and Sensing (S).

In other words, data in the table shows that Intuitive (N) personality indicators are positively associated with openness because they are creative and futuristic-oriented, while practical, grounded, and focused on details are the features of Sensing (S) indicators. To give more meaning to these findings, it is related to basic works on MBTI and the Big Five framework. Such alignment is indicative of theoretical relationship between MBTI’s cognitive preferences and the Big Five’s openness dimension providing a holistic framework of nature or expression of personality traits.

### Motivation and significance

The motivation to determine and study the characteristics of personality arises from the merging of theoretical and practical benefits that are used to understand the complications of human behavior and emotions as well as the study of their cognitive level based on variety of data nature (Song et al., 2023b). This curiosity helps in revealing the facts that help in shaping personality discoveries according to the individual traits and differences involving their personal thoughts and it focuses on the mechanism that controls human societal interaction. The application of natural language processing (NLP) technique personality of various traits of personality traits of individuals. This analysis of personality helps in finding proper individuals for marketing purposes, finding customers regarding products and services (Malik & Bilal, 2024), career counselling (Fatima et al., 2022) and other aspects of personalization (Salminen et al., 2020). The dramatic changes in personality research are supported with advancements in computational techniques and an increase in large data (Naz et al., 2024). The latest AI-based algorithms provide predictive analytics in the study of personality offering improved opportunities in revealing hidden patterns and getting deeper insights. In all this scenario exploring personality traits ensures that it will enhance our knowledge and understanding of real-world challenges in all fields (Christodoulou & Gregoriades, 2023).

This research also addresses the gap in understanding how linguistic features associated with abstract thinking, such as creativity and exploration, contribute to detecting the openness trait. Previous studies have not thoroughly examined how these features manifest in informal language, such as social media posts or personal narratives, which this study explores (William et al., 2023). This study advances the field by utilizing an advanced approach that combines traditional machine learning models, such as support vector machines (SVM) and random forest (RF), with cutting-edge deep learning techniques, including long short-term memory (LSTM) and Transformer-based models. By integrating both word and sentence embeddings, we capture deeper semantic features related to openness, which previous studies have largely overlooked. Sentence embeddings have been applied to capture the detailed semantic characteristics of textual data to predict openness personality traits. That is, the proposed method of advanced embeddings overcomes limitations of other methods based on word-level embeddings or statistical features for utilizing and understanding sentence meaning while preserving context and allowing for highly accurate prediction of outcome. Most importantly, this study examines the potential of sentence embeddings in relationship to MBTI personality prediction. The essence of embeddings is its ability to capture contextual relations within sentences, the features produced by which can improve both traditional and deep learning models (Alsini et al., 2024). Furthermore, our work provides new insights into the specific linguistic patterns that correlate with the openness trait, contributing to more accurate and interpretable personality trait predictions from textual data.

In this research study, our aim is to predict open personality traits from social media posts relying on MBTI dataset. For empirical analysis, the MBTI dataset utilized by applying feature extraction, we used standard features of text features with ML models like Term-Frequency Inverse Document Frequency (TF-IDF) and parts of speech (POS); and for deep learning, word embeddings and sentence embeddings have been used. Traditional ML models such as SVM, decision tree (DT), logistic regression (LR), naïve Bayes (NB), K-nearest neighbor (KNN), and ensemble models like RF, gradient boost (GB), adaptive boosting (AdaBoost), and extreme gradient boosting (XGB) used textual features. Whereas, deep DL including LSTM, and bidirectional long short-term memory (Bi-LSTM) use deep features. In addition, the state-of-the-art Transformers-based model bidirectional encoder representations from transformers (BERT) model is also applied. The results are examined using standard evaluation measures of accuracy, recall, precision, f-measure, area under the curve (AUC), and receiver operating characteristic (ROC). From these results, the proposed features to be effective in term of increasing classifier accuracy for the researchers to consider AI applications in personality traits is an active area of research.

Our main research contributions in this research study are as follows:

- Applying comprehensive feature engineering for personality trait detection including textual features of TF-IDF, POS tagging and word embeddings of word2vec, and GloVe.
- Proposing the advanced approach to derive the semantic features for MBTI openness trait using sentence embeddings as they are particularly useful in retaining the contextual relationships that is necessary for higher accuracy.
- Examination of diverse traditional ML models, and advanced DL algorithms like LSTM and Bi-LSTM and transformer-based model BERT.
- Conducting detailed comprehensive analysis acquiring highest results of 90.52 with accuracy measure with Bi-LSTM coupled with advanced word embedding technique sentence embedded on MBTI dataset as compared to existing literature.
- Achieving overall, the top results with state-of-the-art Transformer-based model Distill BERT, accuracy of 92% to predict the personality trait.

**Figure 2:**
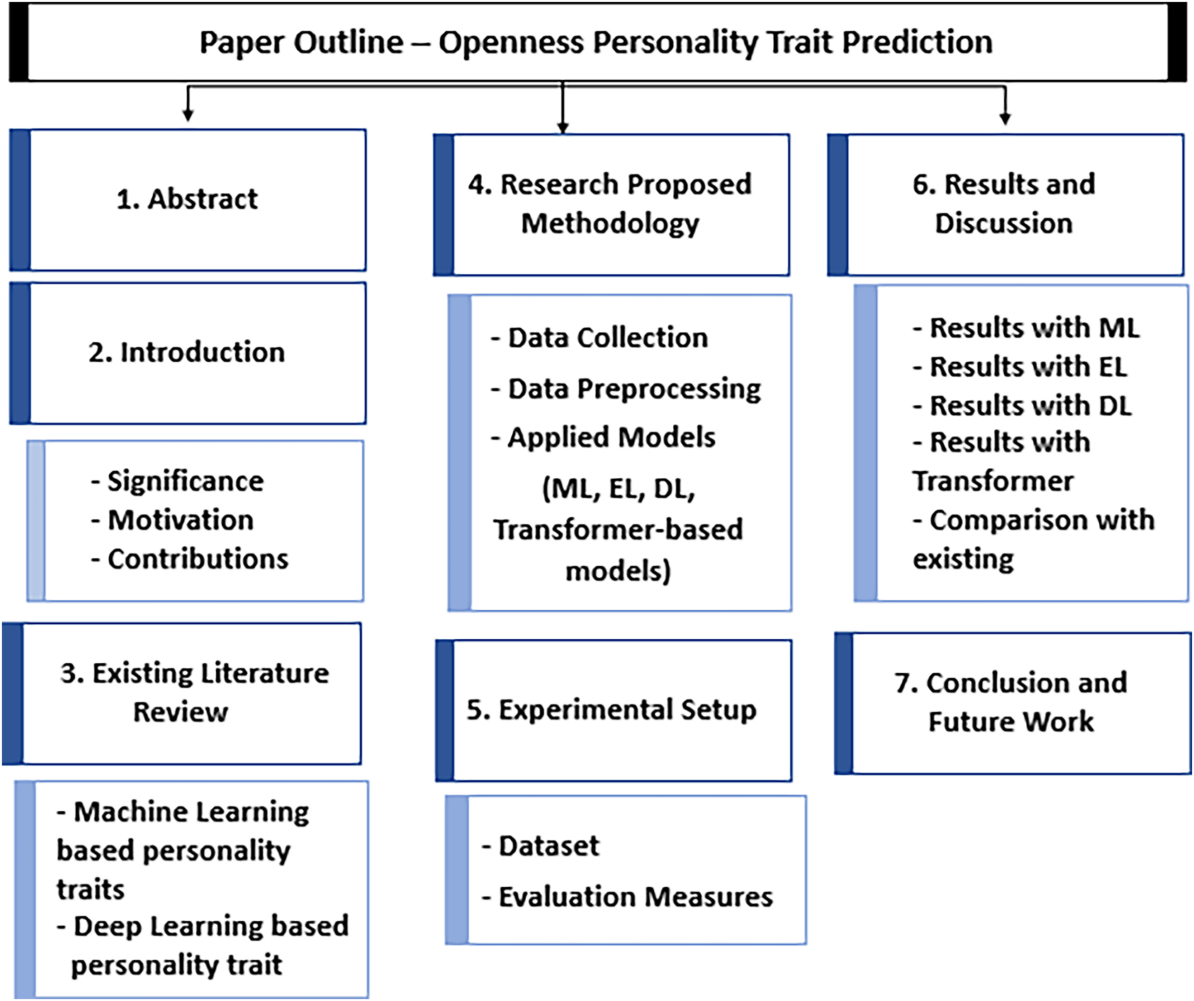
Organization of article.

The rest of the article is structured as follows, shown in Fig. 2: “Related Work” presents the in-depth analysis of existing studies based on AI-based approaches, “Proposed Research Methodology” presents the proposed framework sharing steps of the research methodology, “Experimental Setup” highlights the experimental setup investigating datasets and evaluation measures, “Results and Discussion” presents visualization analysis with results of this study discussed in a comprehensive way, “Conclusion and Future Directions” provides the conclusion of the research along with future directions.

## Related work

Personality trait prediction is a new research area which elaborates on differences in human nature and behavior. Literature on personality prediction in the psychology area helps to analyze individuals by using online platforms. In this study, various ML and DL existing studies have been explored for comprehensive analysis of existing limitations.

### Personality trait prediction using machine learning

The research area still captures change patterns of demographic on human behaviour features and the openness or openness as one of sensibility characteristics. Table 2 defines the summary of existing studies based on ML models. The machine learning model, specifically an SVM, achieved classification accuracy for the traits ranging from 60% to 75%, which is considered optimal results using feature extraction bag of words and TF-IDF. For the openness trait in the hold-out dataset, the accuracy reached 63% (Zhi et al., 2023). NLP-based approach to data-centric prediction of MBTI personality types enhances depersonalized text representation with concerning features generated using sentimental, grammatical, and aspect analyses were applied to each MBTI classifier. The dataset’s representativeness varies, with a significant imbalance in the Intuition *vs*. Sensing trait representation applied with TF-IDF approach following traditional ML models (Basto, 2021). A web platform was developed to collect data for personality assessment using adjectives selection, which was then input into Machine Learning architectures based on GB. Two models were developed: one uses regressors to provide a precise score for the Big Five personality traits, while the other GB employs classifiers achieved F1-score of 83% to provide a categorical output (Fernandes et al., 2020). Another primitive approach developed with the help of binary transformer with feature extraction Term Frequency & Inverse Gravity Moment is suggested to be employed with three dataset—Facebook, Twitter and Instagram. Results of maximum entropy classifier to predict personality trait with up to 83% accuracy (Kamalesh & Bharathi, 2022). One exception approach using clustering, SVM and LR is utilized to predict human behavior using personality trait with the help of my personality dataset to categorize high dimensional feature space (Shah & Modi, 2021). Furthermore, supervised learning (Song et al., 2023a) and unsupervised learning techniques (Ghassemi et al., 2024) based on facial detection also utilized in literature for the prediction of human natures exploring emotions, expressions and signals of individuals, that indicate to personality traits, with the integration of big data methods (Alexander, Mulfinger & Oswald, 2020) can predict a learner’s ability to effectively use self-regulated learning strategies to achieve academic goals with the influence of personality trait levels. The potential for using personality profiles in design of educational experiences and highlights the importance of effective time management and motivation in the self-regulation process (Bruso, Stefaniak & Bol, 2020). The efficiency of various features and regression models for prediction through language centered on psycholinguistic traits that take advantage of the relationships between different personality models (Radisavljević, Rzepka & Araki, 2023). Ensemble learning and SVM are trained with TF-IDF, BoW demonstrates the new classifier for traits prediction utilizing the indicator for personality classification to develop text-based classifiers to categorize dialogues (Ramezani et al., 2022). Twitter hate content is analyzed to identify the level of identification score with the help of users’ profile to classify personality trait by applying ensemble approach RF and XGB comparison with traditional ML models such as SVM, and LR achieving an accuracy of 80% (Perera et al., 2023).

**Table 2:** Analysis of existing studies based on ML models.

Sr. No	Ref.	Year	Models	Dataset	Personality	Features	Result
1	Salminen et al. (2020)	2020	SVM	MBTI	Big Five	Demographic feature ranking	F1: 83
2	Fatima et al. (2022)	2021	BERT	Personality café forum	MBTI	Sentiment analysis	Acc: 64
3	Christodoulou & Gregoriades (2023)	2021	SVM	My-personality	Big five	PCA, LDA	Acc: 72
4	Naz et al. (2024)	2022	Binary-partitioning transformer	Facebook	Big five	Term frequency & inverse gravity moment	Acc: 78
5	Malik & Bilal (2024)	2023	SVM	FMRI	MBTI	Textual feature	Acc: 75
6	Fernandes et al. (2020)	2023	XGB	Pandora	MBTI	LIWC	Acc: 82
7	Shah & Modi (2021)	2023	SVM, XGB, RF	Twitter	Big five	Textual features	Acc: 80

**Note:**

The summary of existing literature based on ML and ensemble models.

### Personality trait prediction using deep learning

Table 3 shows the analysis of existing studies based on DL models. Psychological factors prediction with deep learning approach has vast application using social media to employ word embeddings on textual data require computational resources and large dataset for training emphasizes the successful use in DL models such as CNN, LSTM and RNN (Santos et al., 2023). The recommendation system of using Neural network model also uses the Word2Vec and LSTM layers to analyze textual data to predict personality features (Silpa et al., 2023). Text oriented approach was introduced knowledge graph content to categorize traits separately integration of BERT+NLP using personality and essays dataset achieving an accuracy of 81% (Johnson & Murty, 2023). The works, LSTM for selecting career based on traits prediction (Naik et al., 2022), LSTM CNN (Sujatha et al., 2023), and Bi-LSTM for personality classification using textual content improving with word embedding for text representation achieves 61% accuracy using MBTI dataset (Khattak et al., 2024). To improve accuracy and reliability of the prediction models have XGBoost classifier is used with the BERT transformer model. particularly analyzing user behavior and traits based on social media activities that combine sentiment, grammatical, augmentation, and imbalanced data for better performance for richer text interpretation (Lucky, Roslynlia & Suhartono, 2021). The proposed model effectively combines emotion and personality trait detection tasks, utilizing a model-independent meta-learning framework for efficiency. The learning process significantly improves the approach, leading to root performance in both detection tasks. Leveraging ML and NLP classifications, improving text representation, and feature generation using bidirectional encoder BERT to predict sixteen personality axes by using online social media collected dataset based on MBTI (Rauf, Johan & Aziz, 2023). To optimize DL with limited data by using data fusion techniques and source mapping the essays data was mapped into the big five traits utilizing the CNN-AdaBoost with 59% approach demonstrating its efficacy in automated personality detection by using social media texts (Deilami, Sadr & Tarkhan, 2022). Based on various emotions, to indicate user personality utilizing various type of data (Leekha et al., 2024) enhance the significance of other filed such as for mental health with the help of personality trait predictions the analysis of logic-based approach was conducted using the type indicators that enhance the MBTI by incorporating reasoning factor, allowing for analysis of personality types that can effectively capture the subtleties and complexities of human behavior (Zim, Hanif & Kaur, 2024).

**Table 3:** Analysis of existing studies based on DL models.

Sr. No	Ref.	Year	Models	Dataset	Personality	Features	Result
1	Perera et al. (2023)	2021	BERT	Indonesian Twitter tweets	Big five	Word embeddings	Acc: 71
2	Silpa et al. (2023)	2022	CNN-AdaBoost	Essays	Big five	Linguistic	Acc: 64
3	Santos et al. (2023)	2023	BERT	MBTI	Big five	Word2Vec, BoW	Acc: 78
4	Ghassemi et al. (2024)	2023	RNN-LSTM	MBTI	Five-factor personality	FastText	Acc: 89
5	Alexander, Mulfinger & Oswald (2020)	2023	BERT	Essays	Big five	Knowledge graph	F1: 86
6	Radisavljević, Rzepka & Araki (2023)	2023	LSTM	MBTI	MBTI	LIWC	Acc: 88
7	Ramezani et al. (2022)	2024	Bi-LSTM	Essays	Big five	Word2Vec	Acc: 61
8	Naik et al. (2022)	2024	RNN	MBTI	Big five	Word embeddings	Acc: 78

**Note:**

The summary of existing literature based on ML and ensemble models. LIWC, Linguistic Inquiry and Word Count.

### Limitation of existing studies

In this literature, numerous studies have explored various ML and DL to predict traits from textual data. The majority have primarily relied on traditional features methods such as bag of words or TF-IDF, and with deep features like word2vec and GloVe to present text data. One significant limitation observed in the existing literature on personality traits prediction is the dearth of study leveraging openness personality traits. Therefore, the absence of research by presents a notable gap in current literature. This research focuses on the existing knowledge of how features linked to abstract processing, including creativity and exploration, helping to determine the openness trait. Earlier research has not focused effectively on how such aspects are realized in colloquial text, including Twitter posts and blog entries, which are investigated in this study. Therefore, this study innovates the previous literature by incorporating both the classical ML algorithms together with the new generation’s deep learning algorithms. By incorporating word embeddings with those sentences, we can obtain even more semantically nuanced features concerning openness which have remained largely unexplored in prior work. Moreover, our study offers novel information on the grammatical and lexical features that are connected to the open personality trait, which is instrumental in better and more explainable personality trait estimation from textual data.

## Proposed research methodology

**Figure 3:**
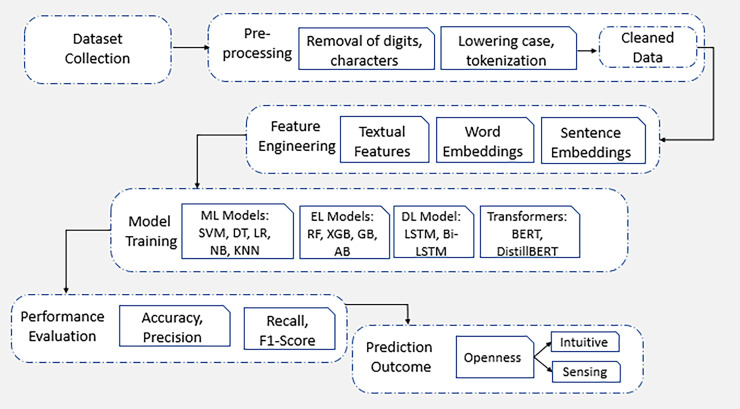
Framework of proposed methodology. The comprehensive analysis of applied proposed framework methodology of this study.

In this section, a comprehensive analysis of features and applied algorithms of ML and DL is presented. Figure 3 shows the proposed framework steps which are followed for research methodology of this study. These analyses further help to understand how personality trait prediction is conducted.

### Data preprocessing

MBTI dataset, which requires meticulous data cleaning and preprocessing to ensure optimal performance of predictive model. Data on MBTI must be preprocessed to make it ready for analysis and ML techniques. The data cleaning process involves several steps:

- Converting all letters to lowercase to maintain consistency and avoid case sensitivity issues.
- Removing links to eliminate irrelevant and unwanted information.
- Elimination of punctuation to simplify the text and focus on the core content.
- Removal of stop-words, such as “and” “the,” and “in,” which do not contribute meaningful information for personality prediction.
- Lemmatization to reduce words to their base or root forms, ensuring the different forms of the same words are treated as single entity.
- Character normalization was applied to standardize characters, handling issues like accented letter ( from “è” to “e”) to ensure uniformity.

**Figure 4:**
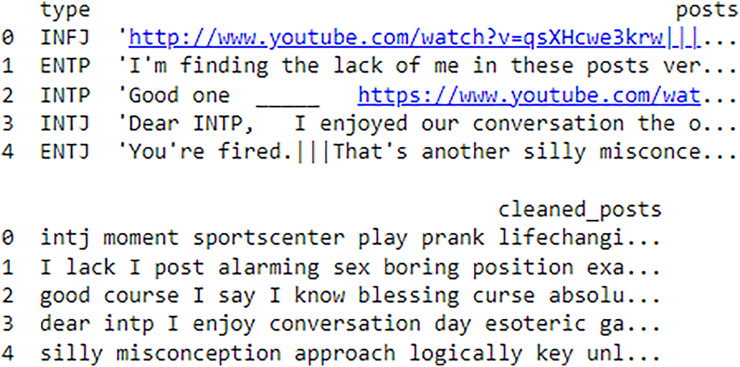
Before and after results of data preprocessing.

Following the data cleaning process as shown in Fig. 4, data pre-processing is proceeded with tokenization step. Tokenization is the process of splitting the cleaned text into individual words or tokens. Once the text is tokenized, the data is preprocessed into a suitable format for ML models.

### Feature engineering

Feature Engineering for text data involves transforming raw text into meaningful features that models can utilize. In this study, textual features such as TF-IDF which quantifies the importance of words in documents, and POS tagging, which identifies syntactic content are conducted for th analysis. Another approach such as, word embeddings to capture semantic relationships by representing words in continuous vector spaces, while sentence embeddings extend this by encoding entire sentences, capturing their broader meaning and context. These methods enhance the model’s ability to understand and predict on textual data (Saeed et al., 2023).

**TF-IDF** used to calculate the significance of a word in a document comparative to a collection of documents by merging two parameters: One is term frequency (TF) and second is Inverse Document Frequency (IDF). By combining these two metrics, through Eq. (1), to ensure that each term’s weight reflects both its frequency within a document providing full normalized form and its significance across the entire *corpus* (Ahmed et al., 2022), providing a balanced measure of term importance. All parameters are defined in Table 4 for understanding.

(1) $$ TF - ID{F_{norm}}\left( {t,d} \right)= \displaystyle{{\matrix{ {\left( {\displaystyle{{log\left( {1 + f\left( {t,d} \right)} \right)} \over {\mathop \sum \nolimits_k log(1 + f\left( {k,d} \right))}} \times log\left( {1 + \displaystyle{N \over {1 + \left| {\left\{ {d \in D:t \in d} \right.} \right|}}} \right) } \right)} \cr \cr } } \over {\sqrt {\mathop \sum \nolimits_{{t}^{\prime}} \left( {\left( {\displaystyle{{log\left( {1 + f\left( {{t}^{\prime},d} \right)} \right)} \over {\mathop \sum \nolimits_k log(1 + f\left( {k,d} \right))}}\times log\left( {1 + \displaystyle{N \over {1 + \left| {\left\{ {d \in D: {t}^{\prime} \in d} \right.} \right|}}} \right) } \right)} \right)} { ^2} }}$$

**Table 4:** List of symbols and their descriptions.

Symbol	Description
${d} \in {D}:{t} \in {d}$	The number of documents in the *corpus*
${N}$	The total number of documents in the *corpus*
${f}\left( {{t},{{d}}} \right)$	The score for a term t in document d
${P}({{t}_1})$	The probability of the first POS tag
${P}\left( {{{t}_{{i}}}{\rm |}{{t}_{{i} - 1}}} \right)$	Probability of transitioning from POS ${t_{i - 1}}$ Tag to ${t_i}$
${P}\left( {{{w}_{{i}}}{\rm |}{{t}_{{i}}}} \right)$	The probability of observing word ${w_i}\;$given its pos tag ${t_i}$
${{v}_{{w}}}$	The vector representation of word w
${\bf C}\left( {\bf w} \right)$	The context of the word w in the *corpus*
${{v}_{{c}}}$	Vector representation of context words
${J}$	Cost function
${V}$	Vocabulary size
${{X}_{{ij}}}$	Co-occurrence of words i and j
${{w}_{{i}}}\;{and\; }{\varpi _{j}}$	Word vectors
${{b}_{{i}}}\;{{and}\; }{\bar { b}}_{{j}}$	Bias terms
${f}\left( {{{X}_{{ij}}}} \right)$	Weighting function
${{v}_{{sentence\; }}}$	Sentence embedding
${{p}_{{wor}{{d}_{{i}}}}}$	Word embeddings of the individua words in the sentence
${{\bf {\cal L}}_{{CE}}}$	Supervised task-specific loss
${{P}_{{teacher}}}^{\tau }{{\; and\; P}_{{student}}}^{\tau }$	Probability distribution from teacher (BERT) and student (DistilBERT) model respectively
${\tau }$	Parameter to control the softness distribution
${KL}$	Distance between models’ probability

**Note:**

The symbols that are used to define the applied equations for computation.

**POS tagging** identifies the sequence of POS tags with the highest chance of yielding the observed words which tagging entails identifying to which grammatical type a particular word belongs (for example noun, verb, adjective *etc*.) Khan et al. (2019) represented as ${w_1}, {w_2}, {w_3}, \ldots \ldots ., {w_n}$ in a sentence, corresponding tags ${t_1}, {t_2}, {t_3}, \ldots \ldots ., {t_n}$, the combine words and tags probability $P({w_1}, {w_2}, {w_3}, \ldots \ldots ., {w_n}$, ${t_1}, {t_2}, {t_3},\ldots \ldots ., {t_n})$ is given by using chain rule computed as in Eq. (2).

(2) $$P({w_i})= P({t_1})\times \mathop \prod \limits_{i = 2}^n P\left( {{t_i}{\rm |}{t_{i - 1}}} \right)\times \mathop \prod \limits_{i = 1}^n P\left( {{w_i}{\rm |}{t_i}} \right).$$

**Word2Vec** is a word embedding technique used to convert words into continuous vector representations, capturing semantic relationships between them. Word2Vec chose word embedding strategies to change the text data map into dense vector representations, capturing semantic relationship among words and sentences based on their co-occurrences in massive textual content corpora. Equation (3) representing the embedding of a word w in the vocabulary is:

(3) $${v_w}= \displaystyle{1 \over {\left| {C\left( w \right)} \right|}}\mathop \sum \limits_{c\epsilon C\left( w \right)} {v_c}.$$

**GloVe** is a method for generating word embeddings by leveraging the co-occurring of words in a data. International phrase-phrase co-incidence records generate embeddings that emphasize each local and global context facts by leveraging the global word-word co-occurrence statistics from a *corpus* is defined as in Eq. (4).

(4) $$J= \mathop \sum \limits_{i,j = 1}^v f\left( {{X_{ij}}} \right)\left( {w_i^T{\varpi _j}+ {b_i} + {{\bar b}_j}- \log {X_{ij}}} \right){^2}.$$

**Figure 5:**
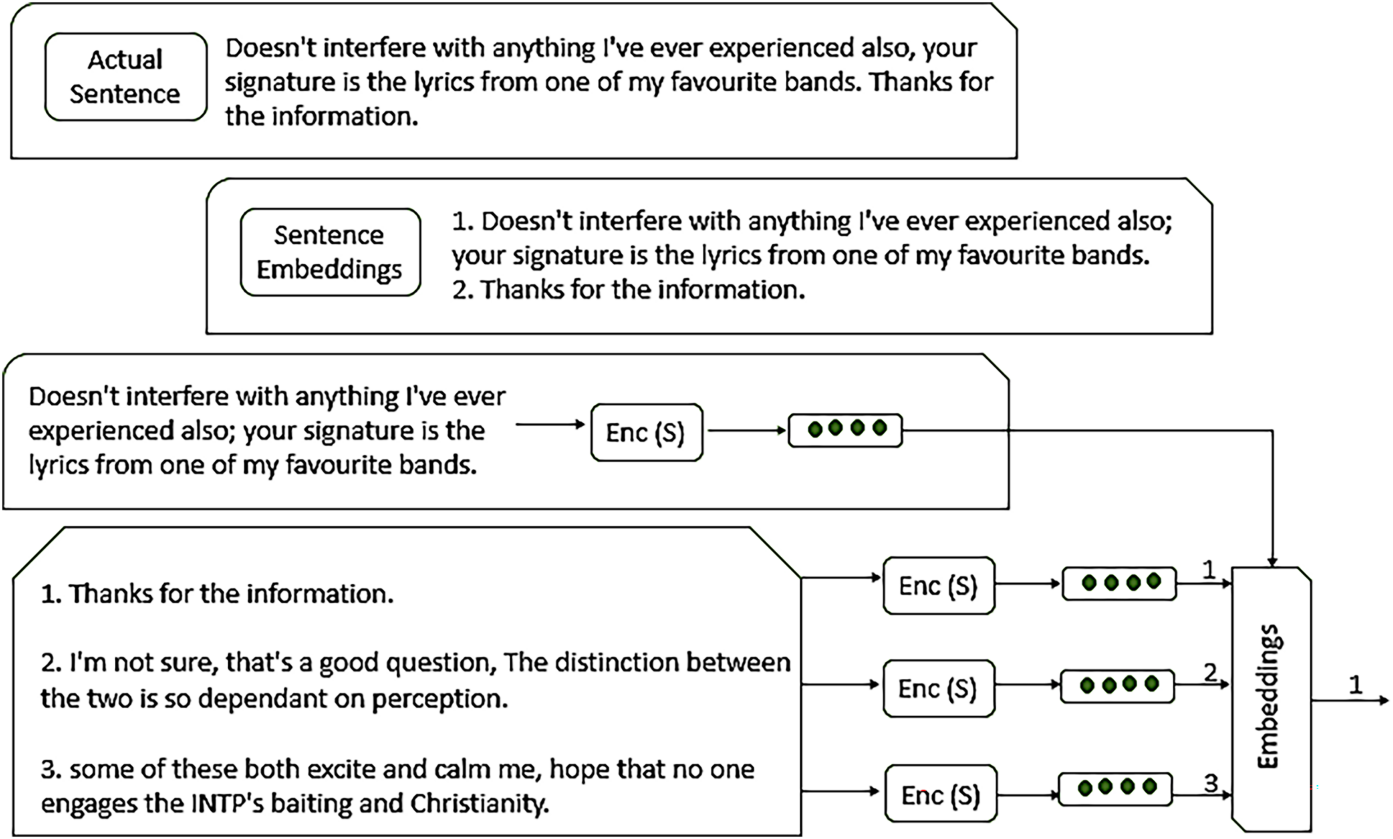
Working of sentence embeddings. The architecture of sentence embedding and how it works to extract features.

**Sentence embeddings** representations in the form of vector notation of whole sentences an effort to capture both the meaning of the sentence and its grammatical phrases. Figure 5 presents the working of embedding, allowing for sentence similarity and comparison by measuring the distance of vectors, as defined in Eq. (5).

(5) $${v_{sentence }} = \displaystyle{1 \over p} \mathop \sum \limits_{i = 1}^p {p_{wor{d_i}}}.$$

### Applied algorithms

We therefore employ a detailed strategy of using computational models for the prediction of openness trait. Prediction using supervised and unsupervised ML and DL exposes how methods of text classification can be grouped according to focus on language. Measured from relatively basic models such as probability models LR, and DT to sophisticated models SVM, and XGB. Likewise in DL, language models are central to NLP tasks, a gets sample of complex framework for developing and interpreting human language. The language has been categorized using varieties such as LSTM, Bi-LSTM and self-attend and Agg transformers, BERT to enable applications such as text summarization, chatbots and other dialogue systems (Ahmed, Khan & Munir, 2024). We applied traditional ML models that provide a baseline for comparison and are typically faster to train and interpret. ML models are effective for initial analysis, helping to identify patterns and relationships in the data. Ensemble models were carried out due to improve prediction accuracy by combining the strengths of multiple models, as ensemble models reduce the variability and lead to better generalization. Advanced deep algorithms are used for their ability to capture long-term dependencies and sequence information in text data, which is crucial for understanding complex personality trait. Transformer-based model is justified for its state-of-the-art performance in NLP tasks, offering deep contextual understanding and higher performance. It’s particularly valuable for capturing subtle patterns in language that are indicative of personality traits. Here is the brief methodology of algorithms conducted in this research.

### Traditional machine learning

Traditional machine learning refers to a class of algorithms and techniques used to perform various tasks such as clustering, regression, and classification (Daud et al., 2023). In the realm of personality trait prediction, our methodology offers a pragmatic approach including SVM, DT, NB, LR, and KNN model, to analyze features extracted from various sources, such as social media post, surveys, and blogs to predict individual personality trait openness with the help of N/S labels from MBTI data presents an insightful exploration of conventional ML approach in personality prediction, models including:

- **Support vector machine** offers a robust framework for predicting personality trait openness by effectively defining boundaries between personality types. SVM high potential in predicting complex relationships within data, that labels exhibit intricate interdependencies with other factors. By leveraging with labels N/S, SVM can discern patterns allowing for accurate classification of openness level. SVM’s ability to identify the optimal hyperplane for separating data points facilitates the extraction of meaningful insights from feature space.
- **Decision tree** utilized for recursive partitioning of features space into the subsets relying on the target feature, aiming to create decision rules that best separate individuals with different openness levels. Each internal node shows a feature, and each leaf node corresponds to a predicted class (*e.g*., high, or low openness).
- **K-nearest neighbor** is the simplest yet powerful algorithm. The predicted class of a data point is categorized by the class label of its closest neighbors in the feature space. KNN is utilized to capture complex correlation in data and control the non-linear decision boundaries.
- **Naïve Bayes** is a probabilistic classifier that features are conditionally independent given the class label. It is computationally efficient and requires minimal data for training to compute the probability of each class features and select the class with the highest possibility as the prediction.
- **Logistic regression** is a statistical model, the possibility of a given input relates to a particular class by using the logistic function to map any real-valued number into the [0,1] range. Classifying N and S labels, LR can effectively model the probability of a given input belonging to one of these classes.

### Ensemble learning models

Ensemble learning models combine multiple learners to improve the prediction accuracy for personality trait predictions models like GB, RF, XGB and AdaBoost to enhance the overall working and predictive accuracy of the system. The essential idea at the back of the ensemble studying is to leverage the collective intelligence of more than one model instead of relying on single model. These techniques aggregate the predictions from different individual models such as decision tree to mitigate overfitting and enhanced generalization (Ishfaq et al., 2022). For instance, random forest creates a multitude of decision trees and average their predictions, reducing variance and improving stability. Gradient boosting, sequentially builds trees to correct errors from previous ones, enhancing model performance. Adaptive boosting adjusts the waves of misclassified instances, focusing on difficult cases. By leveraging ensemble learning, prediction of openness based on NS labels becomes more accurate and dependable, as the combined strength of multiple models can capture complex patterns and interactions better than single models.

### Concurrent deep learning

Deep learning is a subpart of machine learning that works on concepts of neural networks with multiple-layer model complex patterns and representations in data. These models are particularly powerful for task involving large model data and intricate feature interaction. The training of DL models using parallel processing techniques to accelerate training time and improve accuracy refers to the concurrent deep learning models (Khan et al., 2023). Applied algorithm; LSTM networks are type of recurrent neural network used to capture temporal dependencies and long-range correlation in sequential data. In the realm of predicting personality trait openness and the LSTM can effectively model patterns in time series data or sequence of texts such as social media posts or questionnaires that reflect personality traits. LSTM utilized memory cells and gating mechanism to maintain and update information against N/S labels to retain relevant information from earlier in the sequence and use it for current predictions, which is crucial to understand how past behaviors or responses correlate with personality trait. Other model Bi-LSTM network extend LTMS by processing sequences in both forward and backward directions allowing the model to capture content from past and future states (Kazi, Khoja & Daud, 2023). This bidirectional approach increases the ability to understand and understand the patterns in data sets for personality prediction. Bi-LSTM can leverage the full context of data, providing a more comprehensive analysis of start traits like openness manifests through patterns associated with N/S labels.

Integrating these capabilities, LSTM and Bi-LSTM network can effectively model complex patterns and dependencies in personality trait data, leading to more accurate prediction of openness based on MBTI NS label.

### Transformer-based model

**Figure 6:**
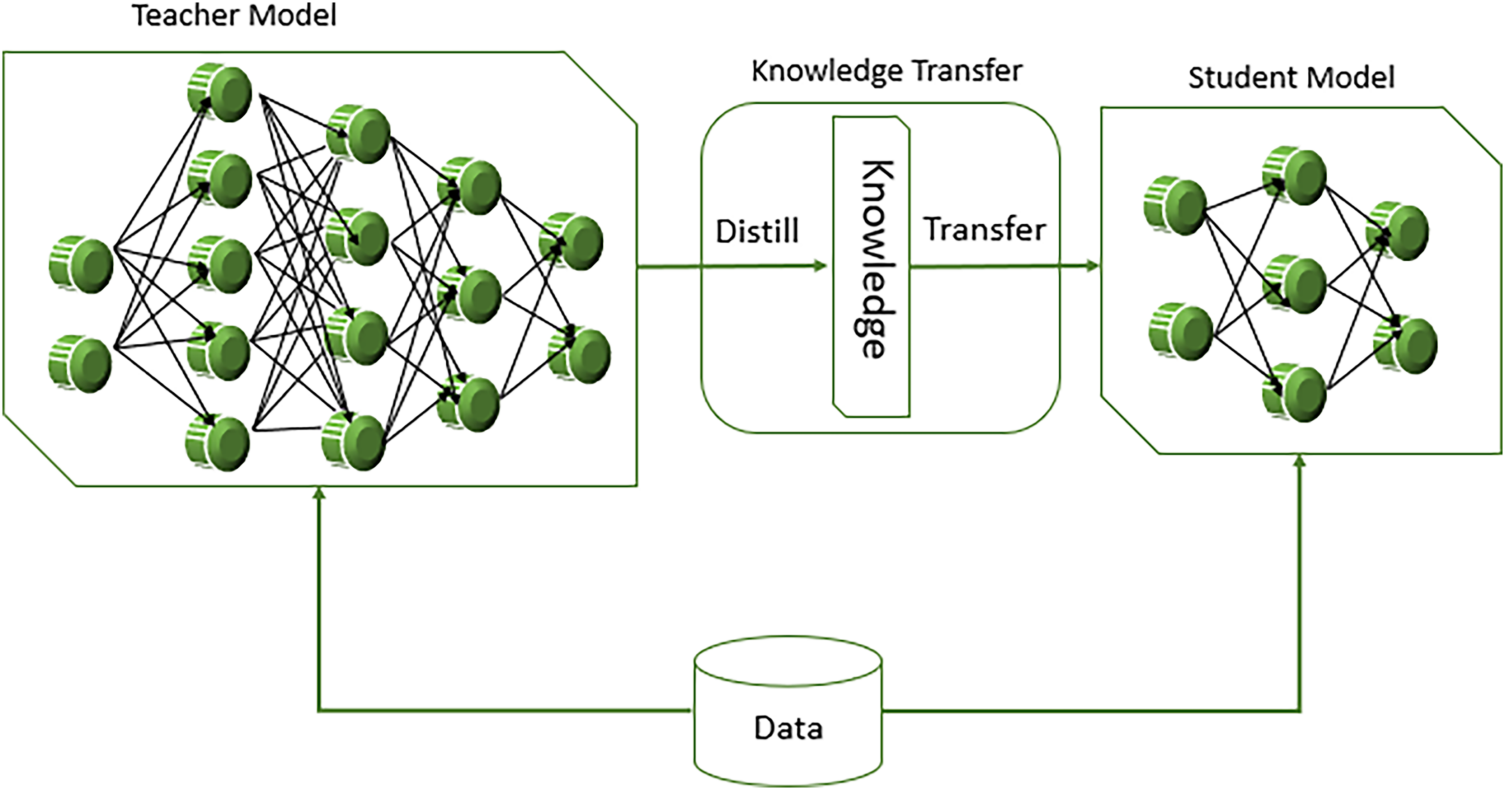
Workings of DistillBERT model in NLP task.

Transformer based models like BERT predict personality trait by leveraging their powerful ability to understand context and semantics in the text data. BERT utilizes transformer architecture, which applies the self-attention mechanism to derive the connection of word from the left and right context (Hayat et al., 2019). This bidirectional approach allows the word to develop a deep understanding of language for predicting personality traits such as openness using MBTI in Intuitive (N) and Sensing (S) labels, BERT fine-tuned on text data where the labels are known. During this fine-tuning process, BERT learns to associate certain linguistic patterns, phrases, and contextual cues with specific personality traits. By processing text data from sources like social media post to identify subtle indicators of traits like openness. Another model, DistilBERT is smaller and faster than pointing BERT with pretraining distilled for a better lightweight version. It does so use a mechanism called “knowledge distillation,” in which a “student” model that’s significantly smaller is exposed to the behavior of a larger “teacher” model—here, BERT. In this way, DistilBERT achieves about 97% of BERT’s language comprehension ability, consuming only 40% of its space and providing 60% faster inference time. Figure 6 displays the working of DistillBERT model.

Thus, DistilBERT is designed to be a lighter version of BERT with only six layers instead of 12 layers in BERT base to retain attention mechanics such as multi-head self-attention and feed forward neural networking. During training phase, not only the student model (DistilBERT) gets trained on the labeled data but also from the softer probabilities generated by the teacher model, as defined in Eq. (6).

(6) $${\rm {\cal L}} = \alpha \cdot {\tau ^2}\cdot KL(P_{teacher}^\tau |{\rm |}P_{student}^\tau {\rm )} + \left( {1 - \alpha } \right)\cdot{{\rm {\cal L}}_{CE}}$$

Such softer probabilities tend to offer deeper insight into the discrimination between classes than just the probability values so aiding the student model to generalize better due to its size. DistilBERT offers a stronger and more efficient option to the BERT model’s true adaptation for the real world’s complicated scenarios and requirements. This makes it particularly effective for complex personality prediction tasks as it can discern the ways in which personality trait manifest in language.

## Experimental setup

This section outlines recommendation for selection of data and then illustrates how data maps from personality trait. It uses Kaggle as an open-source library to compile relevant data. Following the data collection, the text is analyzed with the purpose of selection of the targeted label. When the feature engineering approaches are performed, it is only done on selected labels. Next, models are fitted to predict personality. In Table 5 below, prerequisites are that all codes should be done in Python 3.8.0 while configuration is done on the high-performance local system. Python is a portable high level open-source programming language. While importing libraries that are utilized in our study are: Tensor Flow, pandas, NumPy, Mat plot lib and Scikit—learn respectively. This research utilizes a computer with the following specifications: Intel Core i7-9700 Processor 9th Generation and windows 11 (64 bit).

**Table 5:** Machine requirements.

Parameters	Values
Machine	High performance workstation locally—Intel Core i7
CPU	Intel Xeon Gold 6258R
OS	Windows 11
C U D A	11.2
Cu DNN	V8.1.1
GPU	NVIDIA A100 Tensor Core GPU
Python version	3.8 *via* Anaconda
Libraries	TensorFlow, PyTorch, SpaCy, NLTK, NumPy, Pandas, Scikit-learn
Architectures	Machine learning, Ensemble learning, Deep learning and Transformer based models
Models	SVM, NB, KNN, LR, DT, GB, RF, XGB, AdaBoost, LSTM, Bi-LSTM, BERT
Features	TFIDF, POS, Word2Vec, GloVe, Sentence embeddings

**Note:**

The details of environmental setup on which machine requirements the code is implemented.

### Dataset

In this research study, we selected MBTI (https://www.kaggle.com/datasets/datasnaek/mbti-type) dataset from Kaggle repository. The dataset consists of text data linked to MBTI personality types. Providing a rich source for analyzing the relationship between language use and Personality. The dataset has 8,675 rows and includes a variety of text inputs, such as social media posts, blogs, forum comments, tweets, YouTube links along with corresponding MBTI labels. In research, targeted dataset attends as useful material for studying personality traits, enabling the development, and testing of models that predict MBTI types from distinct personality data, especially in studies we target Intuitive (N) and Sensing (S) labels that is mapped on openness where text analysis and NLP approaches are used to infer personality types from written content.

### Assessment metrics

For the analysis of classification models to predict personality trait, we often employ several performance criteria which are presented in Table 6.

**Table 6:** Equations of evaluation measures.

Sr#	Metrics	Formula	Description
1	Accuracy	$\displaystyle{{TP + TN} \over {TF + FN + FP + TP}}$	To measures the proportion of correctly classified instances out of total number of instances.
2	Precision	$\displaystyle{{TP} \over {TP + FP}}$	It quantifies the accuracy of positive prediction made by the model.
3	Recall	$\displaystyle{{TP} \over {TP + FN}}$	Indicates the model’s ability to capture all positive instances, without missing any.
4	F1-score	$\displaystyle{{2\left( {Precision*Recall} \right)} \over {Precision + Recall}}$	It is useful metric for models with imbalanced classes
5	AUC-ROC	$\mathop \sum \nolimits_{i = 1}^{n - 1} \displaystyle{{\left( {F{P_{i }} - F{P_{i - 1}}} \right)\cdot\left( {T{P_{i}} + T{P_{i - 1}}} \right)} \over 2}$	Quantifies the model’s ability to distinguish between the positive and negative classes across different threshold values.

**Note:**

The applied standard performance measures to evaluate the results of models.

Main performance assessment indicators include accuracy, precision, recall, F1-score and ROC-AUC. are carried out to ensure a comprehensive and fair assessment of the models. These metrics provide a balanced view of how well the models perform across various standard evaluation dimensions, such as overall correctness (accuracy), handling of false positives (precision), capturing true positives (recall), and achieving a balance between precision and recall (F1-score). Moreover, ROC-AUC evaluates the model’s ability to distinguish between classes at different thresholds. Using these standard measures ensures the robustness, comparability, and generalizability of the models across different datasets and applications.

## Results and discussion

**Figure 7:**
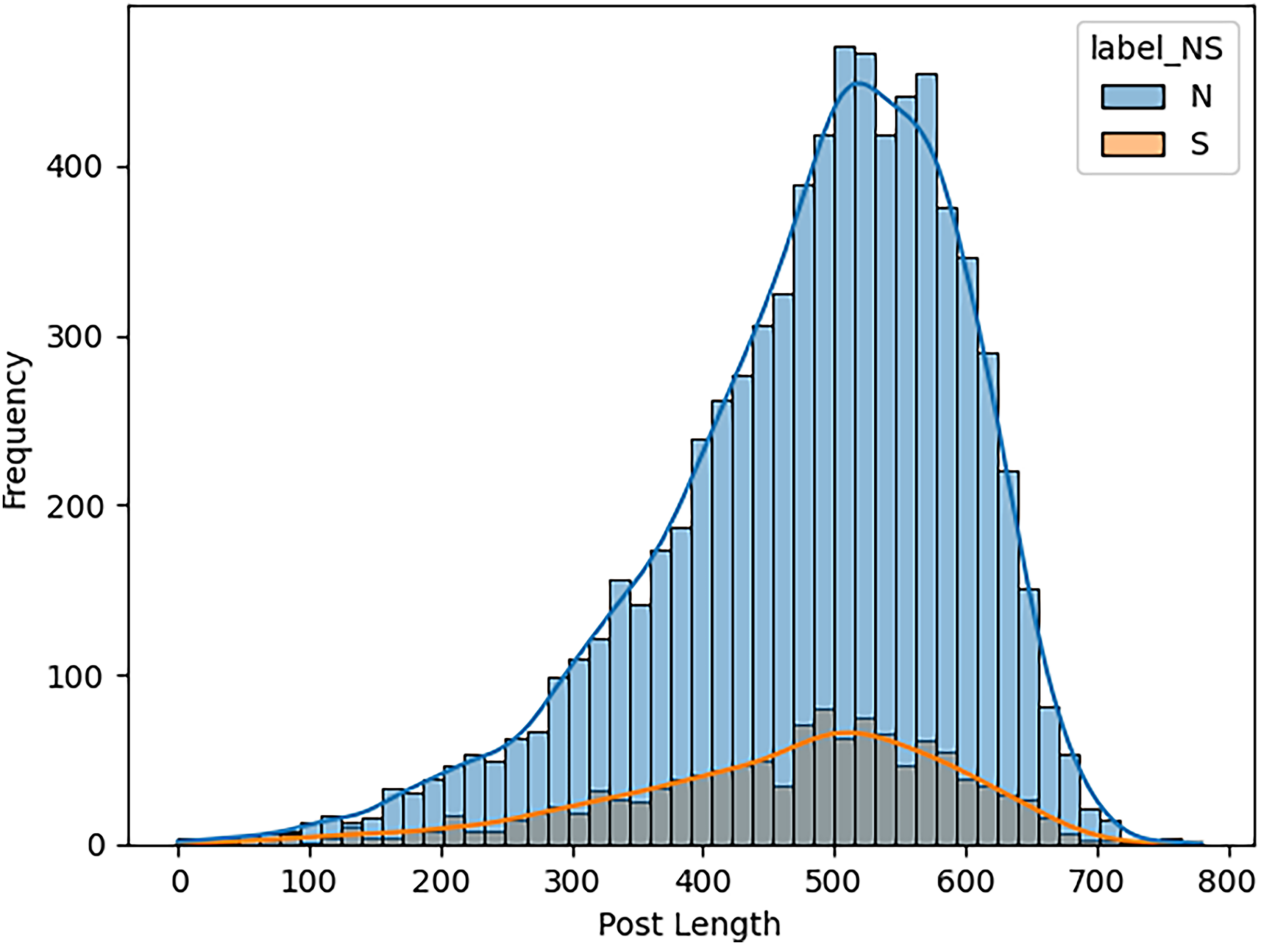
Distribution of posts length by type (N *vs* S). The number or ratio of text in dataset that initiate the level of posts regarding intuitive and sensing.

**Figure 8:**
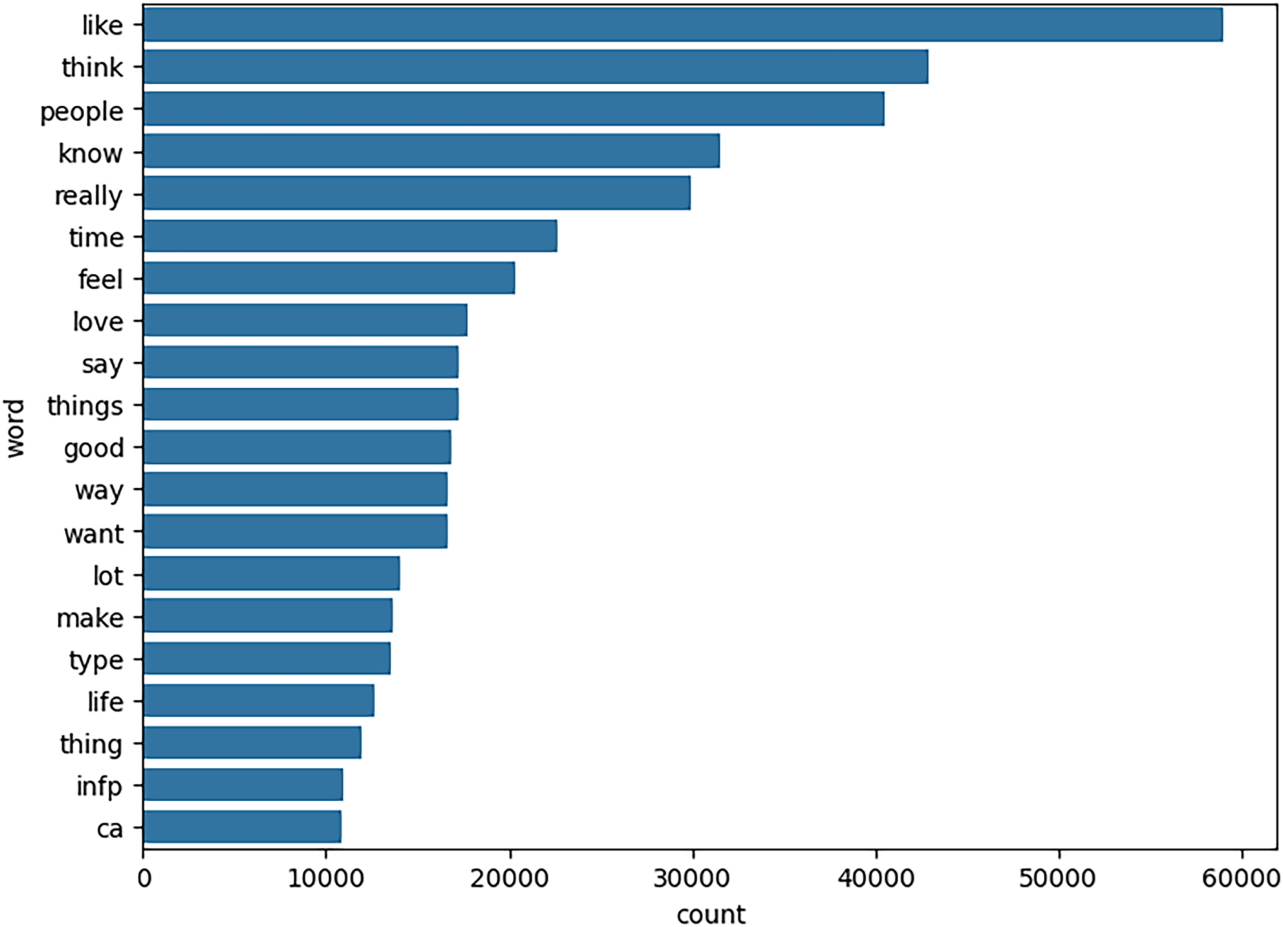
Most common words in labels N and S. The most frequent word based on unigram level that representing the openness personality.

**Figure 9:**
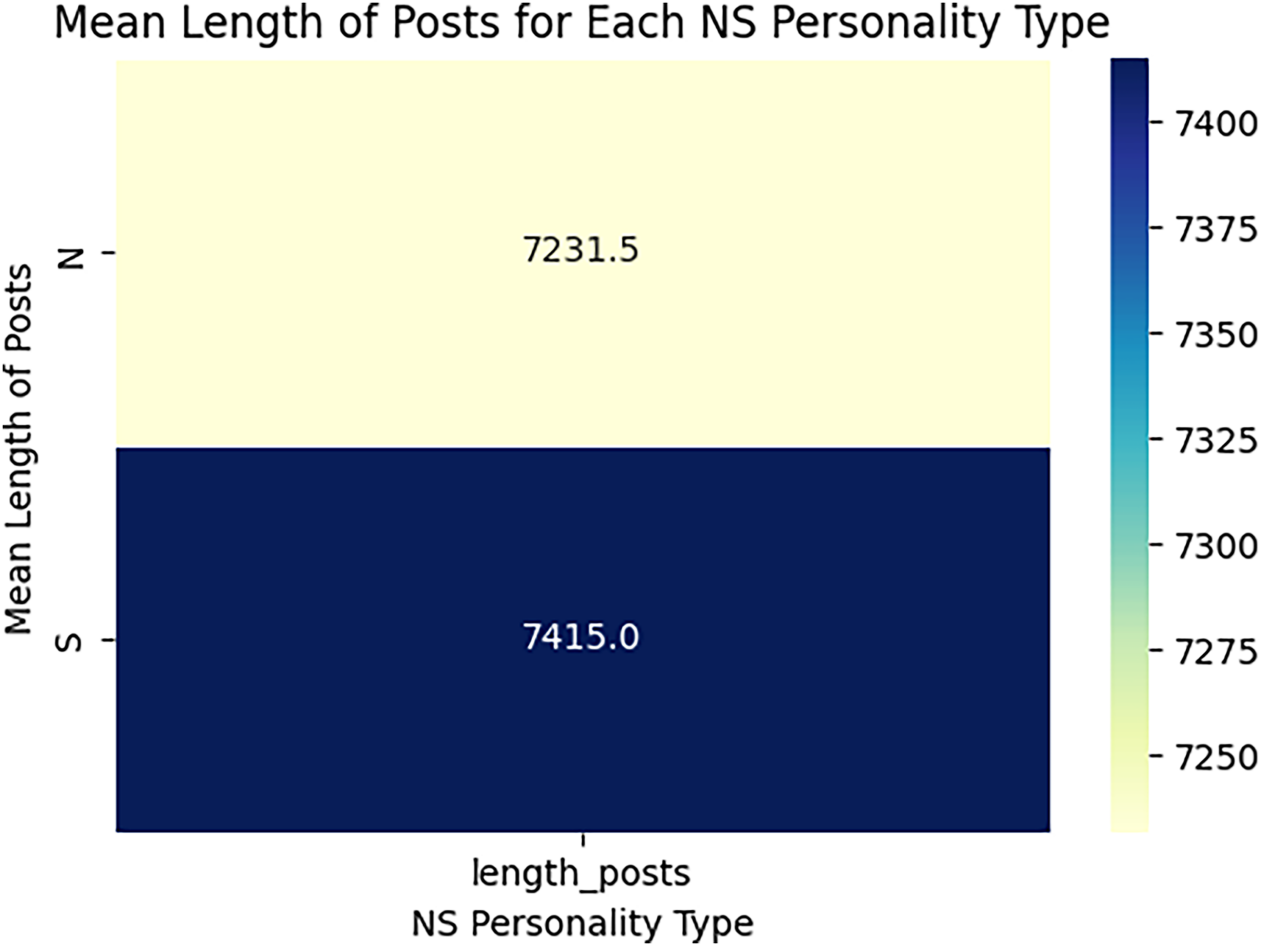
Mean length of posts for label N and S. The average of posts regarding openness dimensions.

When estimating the results of personality traits with the provided MBTI dataset, trait openness corresponds to the N/S axes. We use traditional ML, ensemble models, DL, and transformer model to classify the personality traits with textual features as TF_IDF, POS tagging and word embedding like word2vec, glove and sentence embedded to perform the 80-20 split on personality dataset for the training and testing of model for accuracy of personality trait. This means that 80 percent of the data is used for training and testing sets using 20 percent for testing only so that the testing method checks the result of the model from the unseen data. For exploring and understanding the dataset, with labels intuitive and sensing, through data visualization identify different patterns, trends and differences in language use, post length and vocabulary between two groups. Below we discuss several key diagrams that illustrate various aspects of the dataset. The distribution of posts as shown in Fig. 7 helps in understanding the proportion of data attributed to each label. This figure describes the number of posts in terms of their length for two categories, which are marked as “N” and “S.” This line graph has the post length measured in number of words or characters along the x-axis while the y-axis represents frequency of posting in each length range. The histogram directly shows that, within the category “N”, most posts are shorter compared to the posts in the “S” category, which shows balanced distribution of the posts within a wider range of their lengths. This means that “N” category posts for the most part, would be relatively shorter than the other posts while the “S” category could encompass all forms of post lengths. The distinct vocabulary used by individuals categorized under the targeted label by analyzing the most common words in both labels as shown in Fig. 8. This shows the bar graph of the statistical representation of words found in the posts by frequency of occurrence. “Like,” “think,” “people,” “know”, and other words, shed light on the language and the topics which are most frequently used in the given dataset. These words perhaps be linked to specific personality characteristics or groupings based on the nature of the posts made. The likelihood of these often-occurring words having links to the personnel types characterized in the study needs to be determined. The resulting word distribution could also be a biased distribution especially if the dataset is a skewed distribution. Another illustration in Fig. 9 shows the average meaning of posts indicating differences in communication styles, where one group tends to show its attitude toward content to write longer posts or short. This fig shows particulars of the mean length of items for the different types of NS personality. posts for one personality type have an average length of around 7,415 characters (S), while the other type (N) has a mean length of 7,231.5 characters. This has clearly identified the disparity in the typical lengths of the post, whereby one personality type tends to post more frequently than the other. This difference in post length could be the indication of the variation of the communication pattern between these personality types.

**Figure 10:**
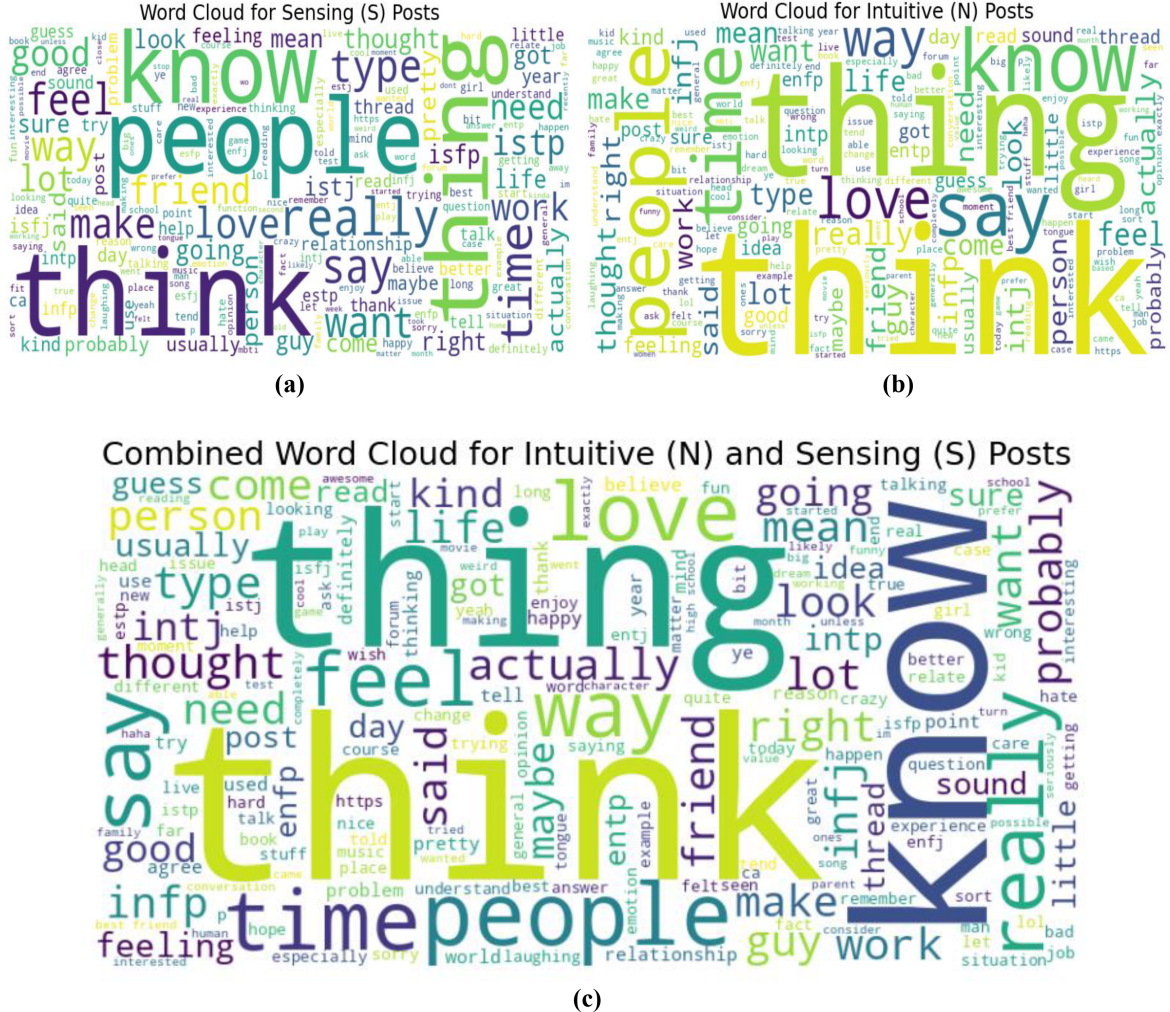
Word cloud of most frequent words in dataset for labels N and S. The word cloud of separate labels and combined to present openness trait.

For qualitative analysis, word cloud as shown in Fig. 10 provides a summary of the most frequently used text data that highlights the core terms used by both groups by reflecting its frequency. As in (a) word cloud of sensing, individuals may focus on present realities, concrete details, and relationships. Words like ‘people,’ ‘think,’ ‘know,’ and ‘feel’ are prominent, reflecting similar concerns with social interactions and personal thoughts. The presence of terms like ‘time,’ ‘friend,’ and ‘good’ suggests that people are meticulous and focused. In (b) for the Intuitive type, the words ‘think,’ ‘know,’ ‘people’ again appear prominently but words like ‘idea,’ ‘thing,’ and ‘love,’ are also more prominent. This suggests a greater emphasis on abstract thinking, future possibilities, and emotional connections. The words that appear frequently in these word clouds align well with characteristic associated with the trait of openness as in (c) which is often linked to creativity, curiosity, and a preference for novelty and variety. Both intuitive and sensing individuals show a concern with thinking and knowing, reflecting an openness to new ideas and a desire for understanding. In combined word cloud words like ‘think,’ ‘know’ ‘people,’ and ‘feel’ are prominent, suggesting both types of personalities engage frequently with thoughts, knowledge, and social interaction. The word ‘thing’ also appears prominently, indicating a focus on object or abstract concepts. These words offer insights into how these individuals approach the world differently based on their focus on abstract *vs* concrete thinking. This focus on thought processes and social connections reflects the broader domain of openness within personality psychology.

### Traditional machine learning models with textual features

The results obtained from applying various ML models to predict openness trait with label N/S using different feature representations offer valuable insights into effectiveness of each approach. Across both TF-IDF and POS tagging representation, the SVM consistently emerges as the top-performing model, showing robustness in handling high-dimensional and noisy textual data. With TF-IDF the highest prediction rate is 89% with SVM models to classify personality openness trait. While other models, such as NB, LR and KNN, demonstrate competitive performance across both features. Similarly, SVM with POS tagging with 86% accuracy highlighting its adaptability to different types of features. Results with machine learning are shown in Table 7.

**Table 7:** Results of traditional ML classifiers with textual feature selection (results in percentage).

Features	Models	Accuracy	Precision	Recall	F1-score
TF-IDF	KNN	86	84	86	81
	NB	86	74	86	79
	LR	87	87	87	83
	DT	84	84	84	84
	SVM	89	88	89	86
POS	SVM	86	74	86	79
	NB	70	74	72	72
	LR	86	74	86	79
	DT	75	75	75	75
	KNN	85	76	85	79

Note:

The results of applied traditional machine learning models such as: SVM, NB, KNN, LR, and DT.

The findings to quantify openness personality traits based on MBTI N/S labels with TF-IDF and POS tagging features explain aspects of model accuracy and feature utility. Such models were able to capture the critical semantic and contextual features because of TF-IDF formula that reduces term frequency in documents by the number of documents. SVM had the highest accuracy of 89% proved high dimensional data handling capability and subtle pattern detection on textual features in addition to high precision, recall and F1 score. LR also showed good results by yielding 87% accuracy with balanced metrics as it works best with TF-IDF, which creates a linear model. The results showed KNN with a good accuracy of 86% verified the usefulness of the system even though it weighed heavily upon the feature scaling factor NB and DT, while receiving a comparatively lower accuracy of 75%, pointed out the inability of the system to handle the high degree of complexity, TF-IDF could not capture.

Meanwhile, the features that based on POS tagging demonstrate moderate performance since here, there is more emphasis on grammatical structures and syntactic roles. SVM was once more identified as the best performer with a mean accuracy of 86%, implying that the method is less sensitive to the types of features used. LR and KNN gave very similar results, suggesting their capability compatible with basic variables. Nonetheless, NB and DT attained 70% and 75% accuracy respectively somewhat lower than the results found by SVM due to the diminished semantic value of POS tagging.

Summing up, the results obtained emphasize the critical role of feature representation in increasing model effectiveness. Hence, TF-IDF turned out to be a better feature engineering technique for personality trait prediction, which contains semantic values and enabled enhanced models such as SVM and LR. The results corroborate the specified methods and stress the significance of integrating optimal feature extraction with highly stable personality prediction algorithms.

### Ensemble learning models with textual features

The provided results demonstrate the performance of ensemble learning algorithms for prediction of trait using TF-IDF and POS features. Ensemble learning techniques such as GB, RF, XGB and AdaBoost, are known for their ability to combine multiple models to improve predictive accuracy. Across both features, GB and XGB achieve the highest accuracy with TF-IDF is 90% for both and with POS tagging 86% and 85% respectively as shown in Table 8. When predicting openness personality traits from the MBTI N/S labels based on advanced feature engineering of ensemble models in combination with TF-IDF, POS tagging, the conclusions are boosted by rigorous and modern machine learning architectures. Specifically, the chosen features based on TF-IDF values yielded high accuracy, reaching 90% on average for both GB and XGB and complex patterns of individual word interactions, while providing similar levels of precision, recall, and F1 scores 89%. These models stand out for their ability to adjust the tree boosting process which improves learning through feature extraction. AdaBoost also emerged well, with accuracy of 89% and metrics very close to each other, this underlines its ability in enhancing poor performers through adaptive boosting. RF seems quite stable yielding an accuracy of 86%, but the F1-Score of 79% shows that there is room for better accuracy in a way that can optimally balance Precision and Recall.

**Table 8:** Results of ensemble classifiers with feature selection (results in percentage).

Features	Models	Accuracy	Precision	Recall	F1-score
TF-IDF	GB	90	89	90	89
	RF	86	88	86	79
	XGB	90	89	90	89
	AdaBoost	89	87	89	87
POS	GB	85	78	86	80
	RF	86	74	86	79
	XGB	87	79	87	79
	AdaBoost	78	66	78	69

Note:

The results of applied ensemble learning models such as GB, XGB, RF and AdaBoost.

The performance of the models with POS tagging features which draw attention to syntactic structures was moderate for the ensemble models. XGBoost outperformed all other algorithms with 87% accuracy while the model remained stable with small contract features that are not semantically richer. Gradient Boosting was closely behind with 85% accuracy proving it can handle a wide variety of features. The accuracy in RF was at 86%, hence it recognized the parameters within the grammatical structure. Nonetheless, AdaBoost turned out to be less accurate with only 78%, along with lower precision, recall, and F1-measure values because of restricted positivity of the POS tagging features.

Such results indicate that the ensemble models have returned best accuracy, especially by GB and XGB which have incorporated richness of semantic TF-IDF for computation. They emphasize the feature representation for boosting model ability and illustrate that TF-IDF offers a better understanding of textual data rather than POS tagging. The results prove the applicability of ensemble learning methods to enhance the accuracy of the algorithm for personality trait prediction. These results highlight the efficacy of ensemble learning techniques such as GB and XGB in capturing intricate patterns within textual data, thereby enabling more accurate predictions of personality trait. The comparison between TF-IDF and POS features for prediction N/S patterns, with TF-IDF features, all models achieve high accuracy scores ranging from 84% to 90%. Particularly the consistently high performance of SVM, GB, XGB and Adaboost models indicating their ability to effectively distinguish between intuitive and sensitive individuals. In contrast, models trained on POS tagging features exhibit lower accuracy and performance metrics compared to TF-IDF based models. While some models, such as SVM, LR and KNN, achieve high accuracy and precision with POS features, others like NB and AdaBoost show lower performance. The next Table 9 presents the hyperparameters of applied models.

**Table 9:** Parameter value settings of machine learning models.

Parameters	Values	Description
Regularization function	Float (1.0, 10.0)	Regularization strength: a lower value makes the model more flexible, and higher value makes it less flexible.
kernel	linear	Specifies the kernel type to be used in the algorithm
N Estimators	100, 200	The number of boosting stages to be run (*i.e*., trees in the ensemble).
learning_rate	0.1	Determines the contribution of each tree to the final prediction
Maximum depth	3	The maximum depth of the individual trees. Larger depths might lead to overfitting
Minimum split	2	The minimum number of samples required to split an internal node.
Weights	Distance, Uniform	Defines how the neighbors are weighted: “uniform” for equal weighting and “distance” for weighted by inverse distance.

**Note:**

The details of machine models parameters with values description used in code for detection.

**Figure 11:**
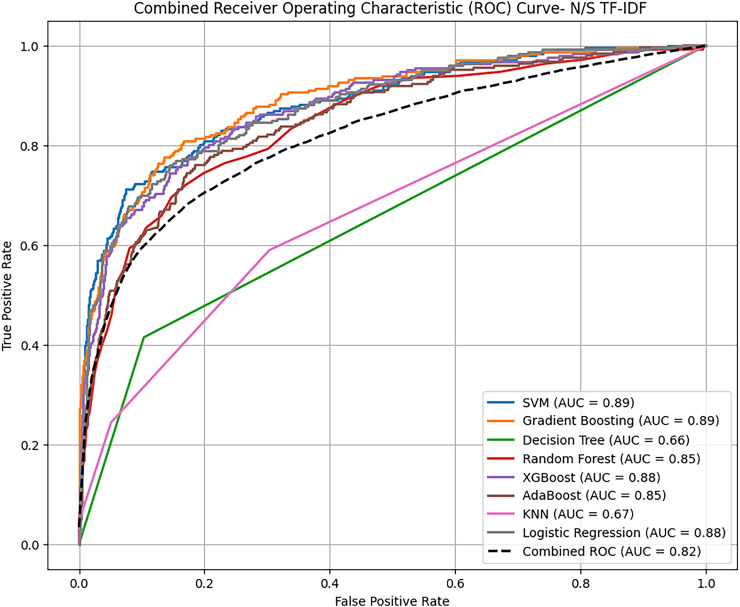
Comparative analysis ROC curve of ML with TF-IDF. The combined analysis of all applied traditional ML and ensemble learning models using textual feature TF-IDF with the help of ROC-AUC curve.

**Figure 12:**
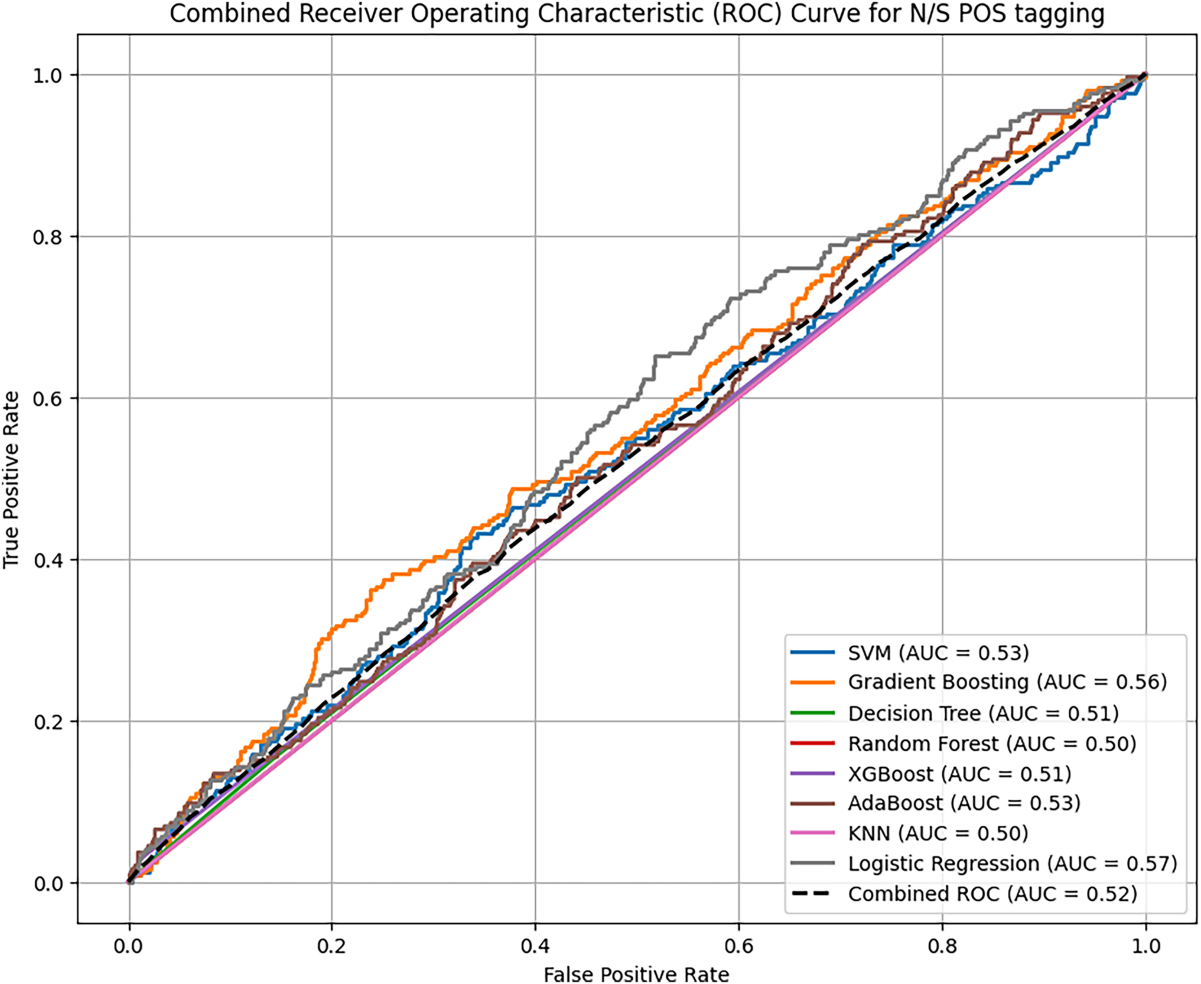
Comparative analysis ROC curve of ML with POS tagging. The combined analysis of all applied traditional ML and ensemble learning models using textual feature POS with the help of ROC-AUC curve.

Based on overall ROC-AUC score produced from feature selection through TF-IDF and POS tagging, it was observed that sentiment analysis model yielded significant performance as depicted in the Figs. 11 and 12. When using TF-IDF with SVM, both GB and XGB yielded high accuracy and f-score as well. This suggests that TF IDF works well in capturing information for discriminating against innate and perceptive characteristics. POS tagged exhibit lower accuracies, with SVM, RF and GB achieving the highest accuracy of 86%, but overall low as compared to TF-IDF. The overall performance of these proposed classifiers with textual TF-IDF and POS feature for personality trait detection are helpful for classifiers to detect traits more accurately. Combined ROC is computed of all models evaluated on dataset for comparative analysis of model performance to identify the superior model results with textual features TF-IDF and POS tagging.

The findings with cross validations show that personality traits based on specific features of ML and ensemble learning models proved as determinative, for example, determining the “openness” personality trait with N and S labels. Different textual features that belong to TF-IDF and POS tagging were applied and tested by cross-validation on various models, as shown in Table 10. Consequently, it was found that GB model provided excellent results for TF-IDF features in terms of accuracy, precision, recall, and F1 score of 90%, 89% and 89% respectively than XGBoost and RF. Most of the models, including the SVM model also gave satisfactory results with similar performance measures. In these models, Naive NB was the worst performing as it struggles when dealing with sparse data features such as TF-IDF.

**Table 10:** Results of all classifiers with feature selection (results in percentage-cross validation).

Features	Models	Accuracy	Precision	Recall	F1-Score
TF-IDF	KNN	87	85	87	84
	NB	86	74	86	79
	LR	88	87	88	85
	DT	86	86	86	86
	SVM	89	88	89	86
	GB	90	89	90	88
	RF	86	85	86	80
	XGB	90	88	90	88
	AdaBoost	89	87	89	87
POS	SVM	86	74	86	80
	NB	70	74	72	72
	LR	86	74	86	89
	DT	83	83	83	83
	KNN	85	77	85	80
	GB	89	88	89	87
	RF	86	87	86	80
	XGB	90	89	90	89
	AdaBoost	89	87	89	87

Note:

Both techniques results of ML and ensemble with cross validation using textual features.

Same trends were noted for POS tagging but the performance rates were slightly lower than the TF IDF feature. GB once more proved to be the best performer yielding an accuracy of 89%; it can be concluded that the package has a good generalization capacity for other complicated features such as POS tags. Another impressive performance was noted with RF and XGBoost models, as ensemble methods are efficient at capturing as many feature relations as possible. For POS features, the results of NB and LR are relatively lower than other models which may indicate that these models do not make proper use of the structural information which is present in POS tagging.

**Figure 13:**
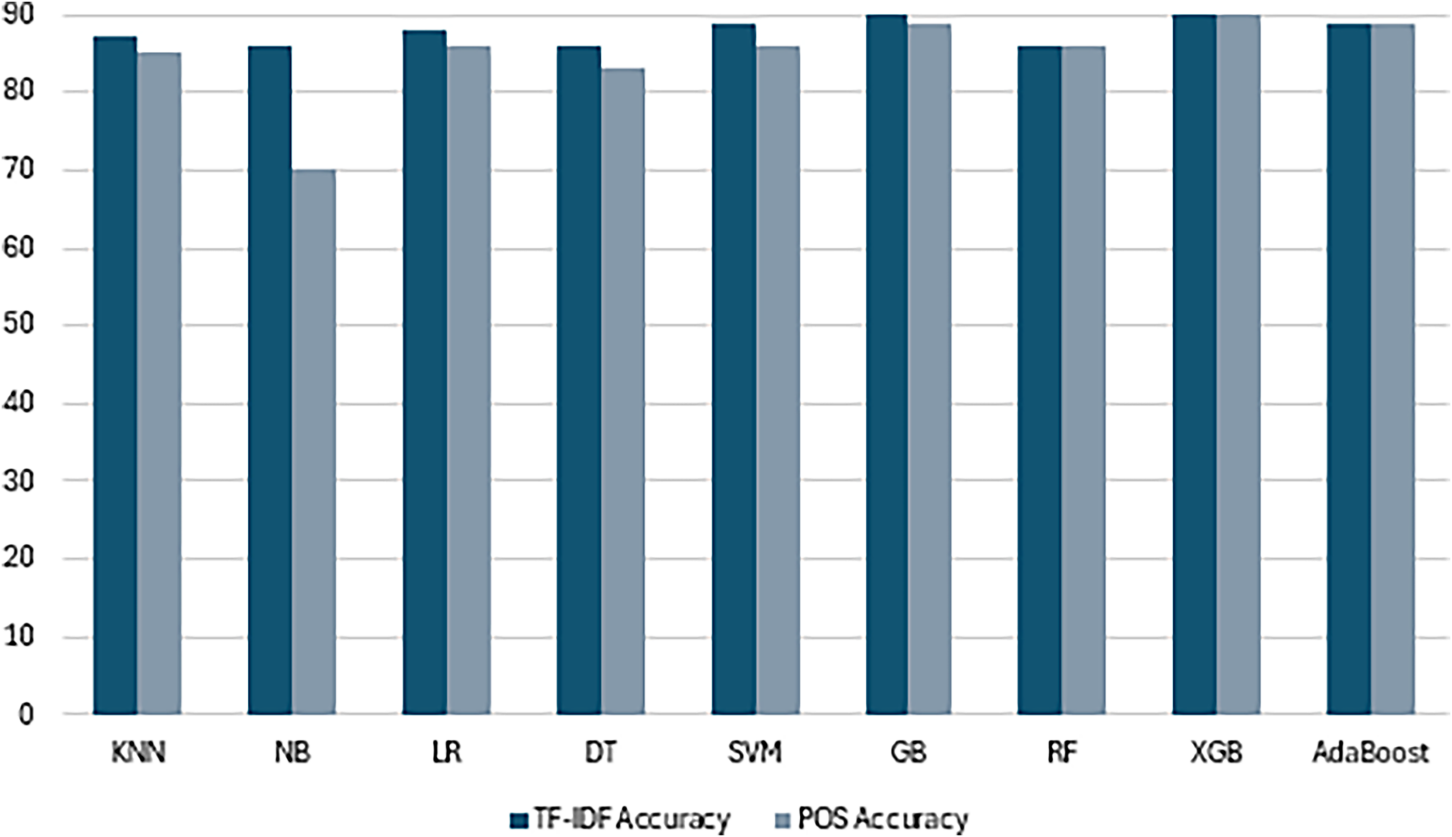
Accuracy measure performance across Textual features. The combined analysis of all ML and EL models using textual features with the help of accuracy measure.

These results provide overwhelming evidence for the efficiency of the choice of ensemble learning models such as GB and XGBoost for predicting the open personality trait based on textual characteristics. Hypothetically, TF-IDF seems to pay better attention to more closely related semantic distinctions than POS tagging does according to all the models, as in term of accuracy analysis shown in Fig. 13. Applying cross-validation made the evaluation reliable and it was clear that these methods promised possibilities to predict openness with N and S labels. These results open the potential for adding new sophisticated functions and applying deep learning methodologies aimed at improving the predictions of personality traits more accurately.

### Deep learning algorithms with word embeddings

When predicting the openness personality traits depending on MBTI N/S labels, deep models and word embedding features demonstrate the advantages of the identified approaches in extracting semantic and contextual data. LSTM and Bi-LSTM computed on the selected features on the test dataset for training to discover the differences between sensing and intuition mapped on the openness trait. The models learn to map the textual features of the corresponding *corpus* in terms of word2vec, glove and sentence transformer. The next Table 11 presents the parameters indicating the architecture of a model: These embeddings can be utilized by the models to arrive an improved understanding of the traits from text, due to the richness of the linguistic information encoded in them. LSTM and Bi-LSTM Model with embedding performance metrics are represented in Table 12 showing with the intention to predict traits on the base of input dataset.

**Table 11:** Hyper-parameter settings of deep models.

Parameters	Values	Description
Input vector size	2,000	Dimensionality of input data that model process, shows number of features after tokenization.
Vocabulary size	1,000	Total numbers of unique tokens, defines the size of embedding matrix
Embedding dimension	128	Size of vector space in which words or characters are embedded.
Unit size	100	Shows the number of units in hidden layer
Number of hidden layers	4	Number of layers between input and output layers.
Activation function	Sigmoid	Applied function to learn complex patterns.
Optimizer	Adam	Used to update the weight to minimize the loss function during training.
Number of epochs	35	Number of complete passes through the entire training dataset.
Batch size	64	Number of training examples used in one iteration
Embeddings	Word2vec, glove and sentence embedding	Representation of words in vector space to capture the linguistic properties of text.

**Note:**

The details of deep models parameters with values description used in code for detection.

**Table 12:** Performance of deep models with word embeddings (results in percentage).

Models	Embeddings	Accuracy	Precision	Recall	F1-score
LSTM	Word2Vec	86.41	85.89	87.01	86.44
	GloVe	84.57	83.78	85.45	84.55
	Sentence embeddings	88.28	87.66	89.14	88.31
Bi-LSTM	Word2Vec	85.50	84.75	86.34	85.48
	GloVe	86.27	85.53	87.11	86.25
	Sentence embeddings	90.52	89.23	88.43	89.21

**Note:**

The results of applied advanced deep learning models such as: LSTM and Bi-LSTM.

In the given experiment of personality classification of intuition and sensing of a person, Word2vec followed with LSTM has recorded an accuracy of 86%. The experiments using GloVe with LSTM have relatively high accuracy in predicting the personality as sensing and intuitional of 84%. The experimental result shows that LSTM when used in Sentence Transformer has an 88% accuracy in classifying personality traits as sensing and intuitional. This proves the effectiveness of Sentence Embeddings for defining deep semantic similarity and contextual correlation in textual information best suited for higher order interpretation of textual data. Bi-LSTM based on Word2vec demonstrates high efficiency in signal and intuitive personality classification with an accuracy of 85%. Likewise, the proposed Bi-LSTM model brought improvements in each case for all the types of embeddings and was the best with the Sentence Embeddings as it offered an accuracy of 90.52%, precision of 89.23%, and F1 score of 89.21%. While Bi-LSTM is handling texts bidirectional, creating sequential structure which can provide both past and future context to any given text making it better in handling of complex languages. This advantage is further enhanced when used together with Sentence Embeddings which has a much higher semantics content representation.

However, Word2Vec and GloVe embedding browsers had high results, where Word2Vec got the maximum accuracy of 86.41% at LSTM and 85.50% at Bi-LSTM while GloVe got a maximum 86.27% at Bi-LSTM. These results suggest using them in representing word-level semantics, but they are somewhat less informative than sentence embeddings, which provide deeper context. Hence, it is also clear that the enhancement of architectures of Bi-LSTM to slightly better performance of GloVe indicates that incorporating advanced architectures in combination with improved feature representations are paramount.

**Figure 14:**
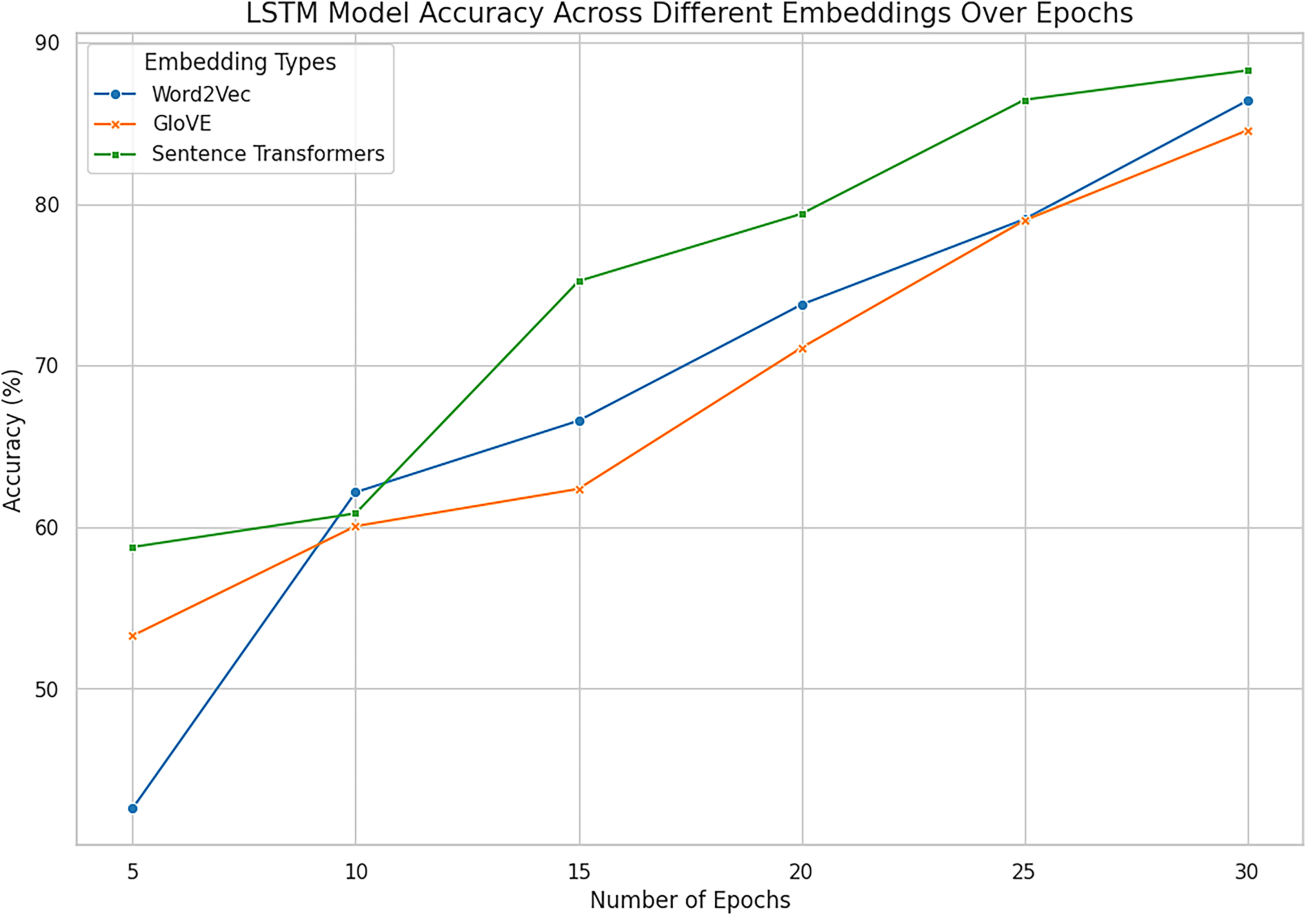
Accuracy across all embeddings with deep model LSTM. The combined analysis of all word embeddings with advanced deep learning model LSTM with the help of line accuracy.

**Figure 15:**
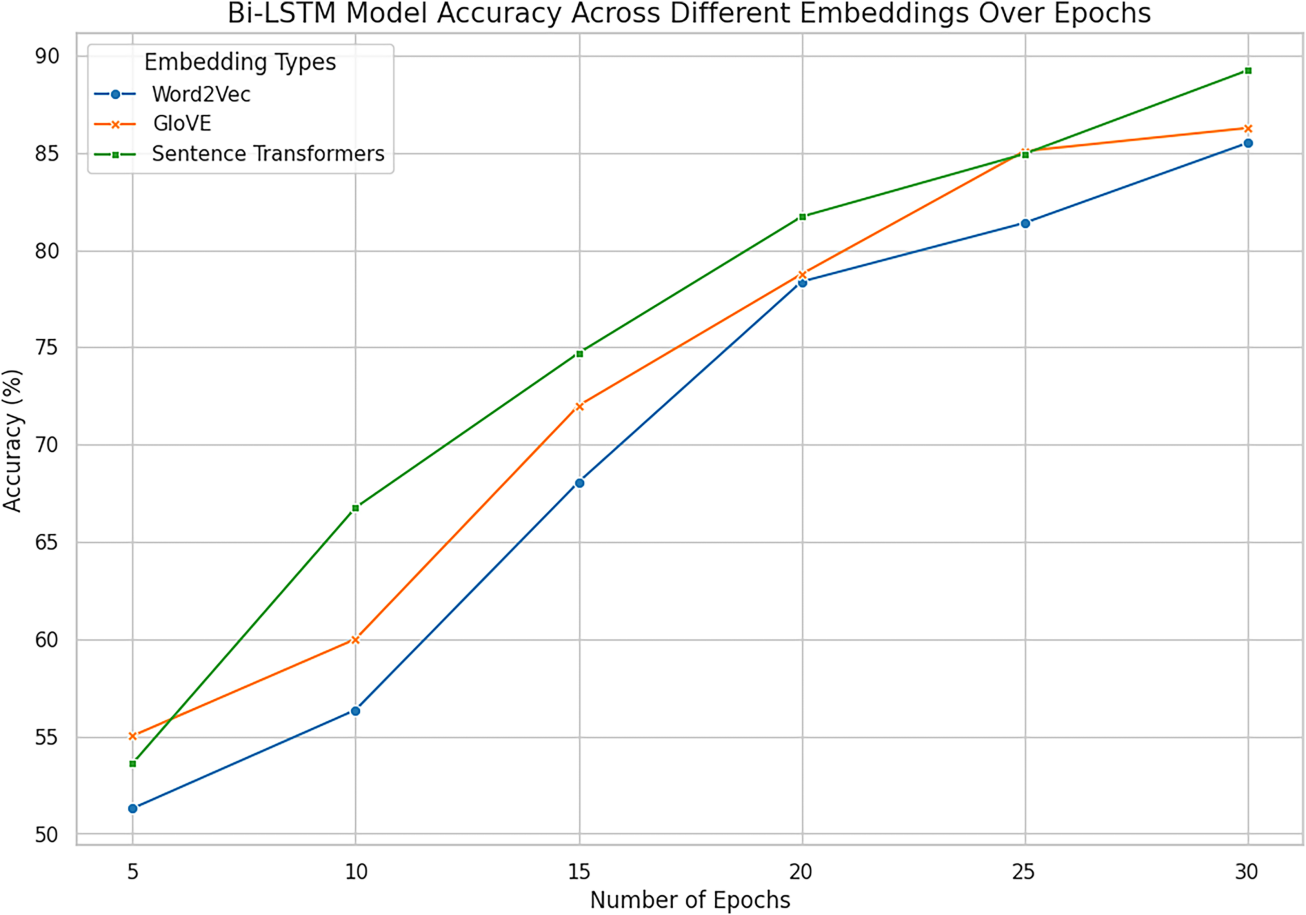
Accuracy across all embeddings with deep model Bi-LSTM. The combined analysis of all word embeddings with advanced deep learning model Bi-LSTM with the help of line accuracy.

Sentence Transformer with Bi-LSTM exhibit promising performance in identifying personality with the accuracy rate of 90.52% as combined outcomes are shown in Figs. 14 and 15. On balance, it can be affirmatively claimed that the Bi-LSTM with sentence embeddings reveals the greatest performance metrics, alluding to its appreciation of relevant and contextually significant features to drive the accurate forecast of openness traits effectively. These results prove the notion of using deep models with complex embeddings as highly efficient for personality prediction problems.

### Prediction with transformer technique

**Figure 16:**
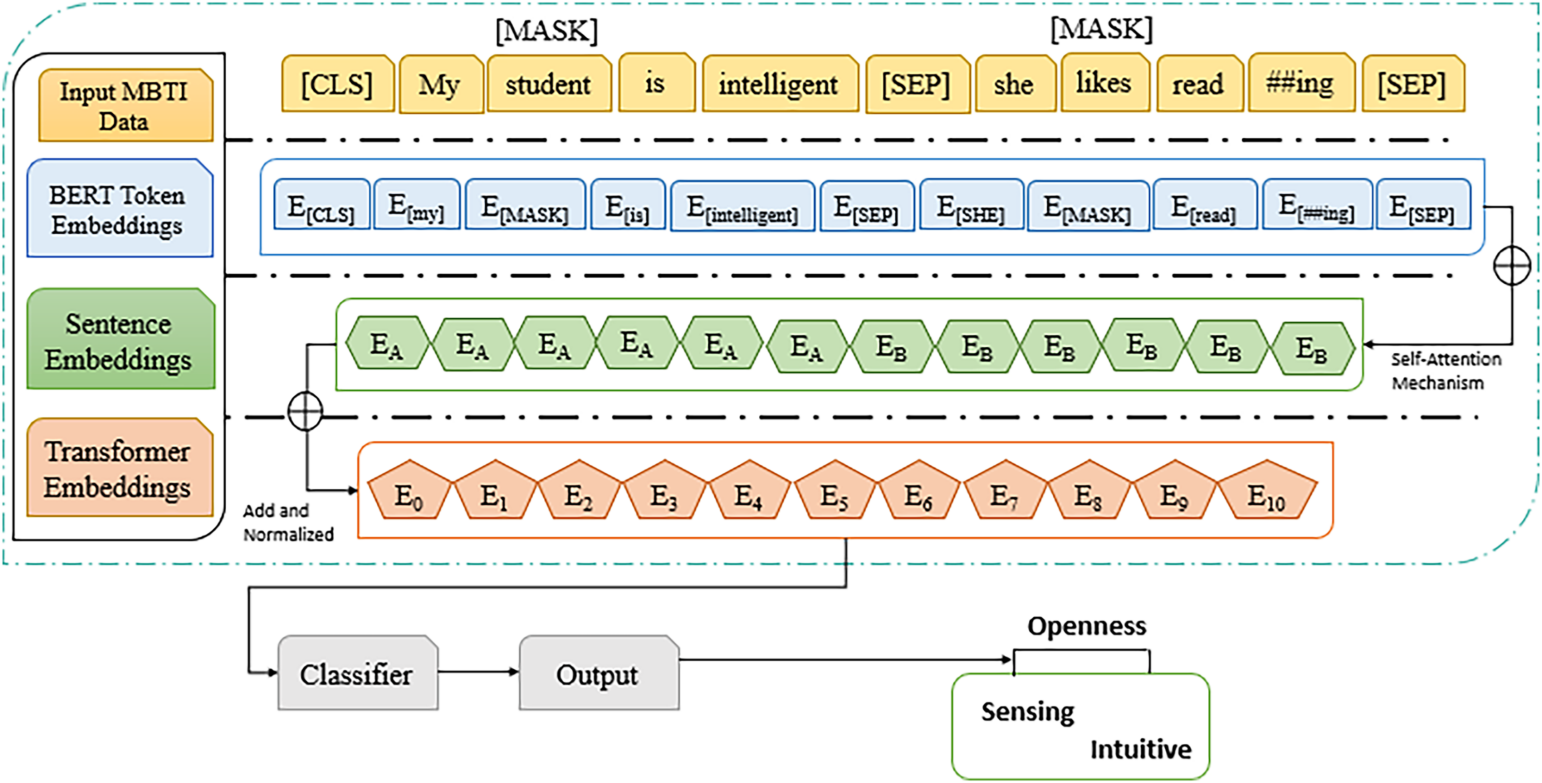
BERT transformer architecture to predict the trait. The comprehensive version applied transformer-based model.

Transformer based model, use of BERT and DistilBERT in our case, which is an attention mechanism that provides high quality representation of text, as working of BERT model shown in Fig. 16. Basically, it has an encoder stack of RNN that encodes the text bidirectional, implying it reads as well as behind to recall context from the words trained on masked language modeling; this predicts tokens that are hidden during training, and sentence prediction determines the relationship between given sentences. This mechanism trained every word in the input in order incorporate improved contextual awareness. In predicting the openness personality traits based on the MBTI N/S labels using the transformer-based models, the machine learning prospects of powerful ML have been clearly shown. The best results were obtained by DistilBERT, that has accuracy of 92.51%, precision of 88.32%, recall of 92.50%, F1-score of 89.21%. This proves how it can work as a good memory-storage device, as well as fast-computing device because it lacks unnecessary hardware components. In large-scale learning personalities’ prediction, DistilBERT has high capability in performance and computational assets, then suitable for the large-scale tasks. Compared to this, BERT gave out an accuracy of 88.24%, the precision of 74.92%, while the recall was 70.55% and F1-score was 71.52%. As can be seen, semantic understanding and contextual representation of the BERT model are a bit lower than those of DistilBERT, but still acceptable. The lower performance in comparison to DistilBERT could be attributed to combinatorial fine-tuning or optimization steps that were employed in training the two because both are transformer-based. The achieved results using proposed model based on variants for prediction with 88.24% accuracy to anticipate trait based on encoded text representation. In comparison to other deep models used for predicting personality traits, the BERT encoder highlighted competitive performance, to classify trait.

These findings provide further evidence of transformer-based models and based especially the DistilBERT, when it comes to identifying and interpreting n-gram patterns and contextual semantics of the textual data. These results further demonstrate that DistilBERT not only performs better but also has sufficient resilience and applicability for tasks that are both precise and fast, reestablishing its position as one of the most effective models in natural language processing for personality prediction.

To compare the effectiveness of the DistilBERT model with BERT model in estimating the openness personality trait. In both cases, SBERT (Sentence-BERT) was used as the encoder because it has been demonstrated to generate accurate sentence embeddings for sentence-level similarity tasks when fine-tuned version of BERT. Table 13 shows the results achieved on both models. Although both the extraction was done in a similar way of feature extraction, DistilBERT outperformed BERT having accuracy of 92% as compared to the accuracy of 88% of BERT. This improvement simply proves that even though DistilBERT is a lighter and computationally efficient model, it can capture complex semantic patterns within the text in a much better way. The knowledge distillation in DistilBERT due to the precise training technique, which guides the model toward learning from the teacher BERT without introducing redundant layers to the network. DistilBERT achieves a perfect balance between performance and efficiency by removing unreactive layers within the transformer architecture and fine-tuning self-attention mechanisms. This means that while training the distilled model the contextual factors were included and when the prediction was made it was much more accurate with other data sets.

**Table 13:** Performance of models with transformer-variant models (results in accuracy (percentage)).

Models	Accuracy	Precision	Recall	F1-score
BERT	88.24	74.92	70.55	71.52
DistilBERT	92.51	88.32	92.50	89.21

**Note:**

The results exploration using state-of-the-art transformer-based models.

These outcomes are especially valuable for the openness trait, which is usually identified by fine differences in the language reflecting such aspects as imagination, creativity or curiosity. The authors credit the higher accuracy of DistilBERT to its better model of these complexities in comparison to the standard BERT model. In conclusion, results approve the selection of DistilBERT model as more accurate and computationally efficient solution for personality trait prediction tasks.

### Overall results performance

Comparing the performance findings across ML, Ensemble, DL, and Transformer-based methods, the research underscores the benefit of sophisticated procedures for determining the openness personality dimension using MBTI N/S labels demonstrate a remarkable performance. The utilization of comprehensive set of models like SVM, DT, RF, LR, KNN, NB, GB, XGB and Adaboost as well as DL models like LSTM and Bi-LSTM and transformer-based model DistilBERT, presenting a novel study apart from no existing work is available to predict trait, from conventional to state-of-the-art approaches are followed in chronological order, overall highest results shown in Table 14. It can be seen from Tables 7 to 8 that among the ML models, SVM achieved an accuracy of about 89% with TF-IDF features which indicate the ability of this model to handle textual data. GB and XGB achieved 90% accuracy with TF-IDF; the result underscores the effectiveness of stacking multiple learners in the process. The additional models, Bi-LSTM with Sentence Embeddings, gave an additional accuracy of 90.52%, thus confirming the deep learning model’s ability to capture semantic and contextual parameters within text. However, the Transformer-based Distil- BERT developed in this study yielded the highest accuracy with a value of 92.51% coupled with the best precision, recall and F1-measures across the approaches used. These results pinpoint the strength of Transformer models in identifying various language patterns and therefore chooses DistilBERT as the proposed model in this study given its high accuracy and efficiency in predicting personality traits.

**Table 14:** Comparisons of all models with proposed models (results in percentage).

Technique	Model	Feature	Results (%)
Traditional ML	SVM	TFIDF	89
	SVM, LR	POS	86
Ensemble	GB,XGB	TFIDF	90
	XGB	POS	87
Concurrent DL	LSTM	Word2Vec	86
		GloVe	86
	Bi-LSTM	Sentence embeddings	90
Transformer model	BERT	Encoder: SBERT	88
	DistillBERT		92

**Note:**

The contribution of this study by comparing the proposed results with all applied features from traditional text to advance word embeddings.

The integration of diverse textual and word embeddings features further enhances the predictive power of the models. With traditional ML models with SVM + TFIDF, subsequently the highest accuracy from all models achieved by GB + TFIDF which shows the remarkable performance of boosting models. Moreover, sentence embedding outperforms the highest accuracy rate of 90.52% with the Bi-LSTM model underscores the effectiveness of advanced deep learning architectures for personality trait prediction. In addition, Transformer-based model, which has gained significant attention in NLP task due to its contextual understanding of language, DistilBERT is employed to capture the linguistic patterns from social media for personality traits prediction with the highest accuracy rate of 92% as compared to existing studies. Overall, these results in accuracy rates highlight the outstanding results of proposed methods with model potential for exploring more domain for research purposes in different ways for the prediction of personality trait from textual content.

The discussion of results focuses on the possibility of applied methodologies to predict the open personality trait: Intuitive (N) and Sensing (S). The results substantiate the hypothesis by revealing that advanced text analysis can accurately analyze a multifaceted personality trait “openness.” Only those models that incorporated both textual features and contextual representations proved to be robust at distinguishing the trait related linguistic features. These patterns suggest that the Intuitive individuals use conceptually associative thought processes when it comes to language, while the Sensing, individuals would be inclined toward sensorimotor language use.

These subtleties were especially important in restructuring by including new advanced word embeddings and transformer techniques for increased prediction performance. Primordial analysis of text data was given by TF-IDF for word count and word importance; on the other hand, contextual embeddings inclusion of sentence embeddings made it more possible to gain further in depth understanding of words’ semantics. Such findings confirm that personality characteristics can indeed captured from the patterns of language, and these findings showed huge informative value for psychological diagnostics, communications with specific goals, and subsequent individualized interventions. The predictive accuracy in identifying openness traits shows the possibility of natural language processing in interpreting and characterizing human actions.

One limitation of this study is that it focuses exclusively on the prediction of Openness personality trait. While Openness is a critical dimension of personality trait, there are other equally important traits that also significantly contribute to the overall understanding of individual differences in behavior. Moving forward, we will explore this research by adding more traits using LLM techniques, thereby enhancing the applicability of and robustness of our findings across the full spectrum of personality dimensions.

### Comparison of proposed results with existing studies

For further enhancement of the predictive power of the models, diverse textual features such as TF-IDF, POS tagging, and word embedding including Word2Vec, GloVe, and advanced technique sentence embeddings are utilized in this research. Moreover, TFIDF+GB, XGB achieves the highest accuracy of 90% to predict the trait. However, we implement the advanced feature sentence embedding with approximately achieving the high accuracy rate of 90.52% with the Bi-LSTM model underscores the effectiveness of advanced deep learning architectures to capture the linguistic patterns from social media for personality traits prediction with the highest accuracy rate as compared to existing studies. Overall, this considerable enhancement of the results proves the efficiency of the proposed approaches over existing methodologies, as evidenced by Table 15, indicating the prospects of the work for further development of the research of personality trait prediction from social media content.

**Table 15:** Comparisons of all models with existing studies (results in percentage).

Ref.	Year	Model	Features	Results
Amirhosseini & Kazemian (2020)	2020	XGB	TF-IDF	86
Sirasapalli & Malla (2023)	2023	BERT	Word embeddings	84
Leekha et al. (2024)	2024	SVM	TF-IDF	86
Proposed—2024		Bi-LSTM	Sentence embeddings	90
		DistillBERT	Default	92

Note:

The contribution of this study by comparing the proposed results with existing studies.

## Conclusion and future directions

The research contributes to growing a body of knowledge on the intersection of personality psychology and digital behavior analysis that holds substantial implications for personalized user experiences, targeted marketing strategies and broader psychological studies. This study explores the intricate relationship between social media activities to predict Openness as a trait. The integration of advanced ML and DL models with MBTI framework has provided a robust method for analyzing and predicting traits from UGC. Our findings reveal the comprehensive preprocessing techniques such as tokenization, lemmatization, stop words, URL, emojis removal has enhanced the quality and relevance of the data, leading to more precise predictions. The main purpose of proposed features is to increase the classification accuracy of classifiers and detect traits efficiently. To evaluate the performance of ML classifiers, ensemble models, Transformer-based model, and DL models on proposed feature set to find the most promising features of trait. Performance of all classifiers is measured by evaluation metrics. The results across various personality traits and modeling approaches exhibit distinct patterns in predictive performance. TF-IDF features consistently yield strong results across traits with 89% accuracy using the SVM model, 90% accuracy with GB model and advance sentence embedding achieve accuracy of 90.52% with Bi-LSTM model, and among all, transformer-based model DistilBERT achieved highest performance of 92% accuracy as compared to all other approaches, highlighting their effectiveness in capturing relevant textual information for personality prediction tasks.

One limitation of this study is that it focuses exclusively on the prediction of Openness as a personality trait. While Openness is a critical dimension of personality, there are other equally important traits that also significantly contribute to the overall understanding of individual differences in behavior. Moving forward, we will explore this research by adding more traits using LLM techniques, thereby enhancing the applicability of and robustness of our findings across the full spectrum of personality dimensions.

There is exciting future work, which involves extending the set of personality traits and the linkage to other psychological theoretical models and AI techniques for personality prediction. Enlarging personality prediction to languages such as Urdu and Pashto presents difficulties due to data availability, linguistic complexities, and cultural differences, and how these variables affect personality prediction across languages. English language datasets are used to predict as prevalent resource, but low resource data is still unavailable. Creating strong models that can be generic across several languages and cultural conditions is a significant task. The consequences of this research show how machine and deep learning has vast promise in the collaboration of the psychology domain to predict personality traits and cover the technique for further advancement in this exciting and vital field, offering valuable fields for analysis as future research and practical applications in personality assessment and behavior analysis.

## Supplemental Information

10.7717/peerj-cs.2781/supp-1 Supplemental Information 1 Myers-Briggs Type Indicator (MBTI) Dataset. The dataset contains posts from individuals along with their respective MBTI personality types. The MBTI types are composed of four dichotomies: Introversion (I) *vs*. Extraversion (E), Intuition (N) *vs*. Sensing (S), Thinking (T) *vs*. Feeling (F), and Judging (J) *vs*. Perceiving (P).

10.7717/peerj-cs.2781/supp-2 Supplemental Information 2 Openness Personality Trait Prediction-Code. In this code, we applied various machine learning (ML) models to predict personality traits using text features. The Support Vector Machine (SVM) model, leveraging text features, achieved an accuracy of 89%. Ensemble models, including XGBoost (XGB) and Gradient Boosting (GB), were trained using TF-IDF features and resulted in accuracies of 86%. Moving to deep learning models, the Bidirectional Long Short-Term Memory (Bi-LSTM) network, utilizing sentence embeddings, attained an accuracy of 90%. Finally, the Transformer-based model achieved 88% accuracy, further showcasing the effectiveness of advanced NLP techniques in personality trait detection.

10.7717/peerj-cs.2781/supp-3 Supplemental Information 3 The description of applied model in our work. By applying advanced deep learning models including transformer-based model, the comparison is shown using Shallow Machine learning models and Ensemble Models.

10.7717/peerj-cs.2781/supp-4 Supplemental Information 4 The evaluation criteria of our model progress.

10.7717/peerj-cs.2781/supp-5 Supplemental Information 5 The focused point in our study to predict personality trait using online textual content on social media platforms.

10.7717/peerj-cs.2781/supp-6 Supplemental Information 6 Reproduceability: description of implementation with results overview.

## Additional Information and Declarations

### Data Availability

The following information was supplied regarding data availability:

The code is available in the Supplemental Files.

The code is available at GitHub and Zenodo:

- https://github.com/Anamt761/Personality-Code.

- Anam. (2024). Anamt761/Personality-Code: Personalityv0.1 (Personalityv0.1). Zenodo. https://doi.org/10.5281/zenodo.13853275.
