# Supplementary material for: Using transformers and Bi-LSTM with sentence embeddings for prediction of openness human personality trait
Source: PeerJ Comput Sci. 2025 May 22;11:e2781. doi: 10.7717/peerj-cs.2781 (PMC12190728; doi:10.7717/peerj-cs.2781)
Supplement: Supplemental Information 3 — By applying advanced deep learning models including transformer-based model, the comparison is shown using Shallow Machine learning models and Ensemble Models. [file peerj-cs-11-2781-s003.docx]

**Description of models**

We conduct a comprehensive approach using computational models for predicting openness trait. Investigation with ML and DL models shows how different methods including supervised, unsupervised, and hybrid approaches, can categorize text classification by focusing on language. These models range from conventional models like LR and DT to advanced ones like SVM and XGB. Similarly, in DL, language models play a critical role in NLP tasks, showing sophisticated framework for generating and understanding human language. Models like LSTM, Bi-LSTM, and transformer models, like BERT, have categorized the language to enable applications as text summarization, chatbots and dialogue systems.

- **Justification for model type used**

We applied shallow ML models that provide a baseline for comparison and are typically faster to train and interpret. ML models are effective for initial analysis, helping to identify patterns and relationships in the data. Ensemble models were carried out due to improve the prediction accuracy by combining the strengths of multiple models, as ensemble models reduce the variability and lead to better generalization. Advanced deep algorithms are used for their ability to capture long-term dependencies and sequence information in text data, which is crucial for understanding complex personality trait. Transformer-based model is justified for its state-of-the-art performance in NLP tasks, offering deep contextual understanding and higher performance. It’s particularly valuable for capturing subtle patterns in language that are indicative of personality traits. Here is the brief methodology of algorithms conducted in this research.

- **Assessment metrics (justification)**

When evaluating classification models for predicting personality traits, several performance metrics are commonly used, as shown in table 4.

**Table 4:** Equations of Evaluation Measures

| Sr# | Metrics | Formula | Description |
| --- | --- | --- | --- |
| 1 | Accuracy | $\frac{TP+TN}{TF+FN+FP+TP}$ | To measures the proportion of correctly classified instances out of total number of instances. |
| 2 | Precision | $\frac{TP}{TP+FP}$ | It quantifies the accuracy of positive prediction made by the model. |
| 3 | Recall | $\frac{TP}{TP+FN}$ | Indicates the model’s ability to capture all positive instances, without missing any. |
| 4 | F1-score | $\frac{2(Precision*Recall)}{Precision+Recall}$ | It is useful metric for models with imbalanced classes |
| 5 | AUC-ROC | $\sum_{i=1}^{n-1} \frac{\left( {FP}_{i}- {FP}_{i-1} \right). \left( {TP}_{i}+ {TP}_{i-1} \right)}{2}$ | Quantifies the model’s ability to distinguish between the positive and negative classes across different threshold values. |

Where TP, TN FP, and FN stand for True Positive, True Negative, False Positive and False Negative, respectively.
