# Supplementary material for: Using transformers and Bi-LSTM with sentence embeddings for prediction of openness human personality trait
Source: PeerJ Comput Sci. 2025 May 22;11:e2781. doi: 10.7717/peerj-cs.2781 (PMC12190728; doi:10.7717/peerj-cs.2781)
Supplement: Supplemental Information 4 [file peerj-cs-11-2781-s004.docx]

- **Assessment metrics (justification)**

Standard performance evaluation measures, such as accuracy, precision, recall, F1-score, and ROC-AUC, are applied to ensure a comprehensive and fair assessment of the models. These metrics provide a balanced view of how well the models perform across various dimensions, such as overall correctness (accuracy), handling of false positives (precision), capturing true positives (recall), and achieving a balance between precision and recall (F1-score). Additionally, ROC-AUC evaluates the model’s ability to distinguish between classes at different thresholds. Using these standard measures ensures the robustness, comparability, and generalizability of the models across different datasets and applications.

When evaluating classification models for predicting personality traits, several performance metrics are commonly used, as shown in table 4.

**Table 4:** Equations of Evaluation Measures

| Sr# | Metrics | Formula | Description |
| --- | --- | --- | --- |
| 1 | Accuracy | $\frac{TP+TN}{TF+FN+FP+TP}$ | To measures the proportion of correctly classified instances out of total number of instances. |
| 2 | Precision | $\frac{TP}{TP+FP}$ | It quantifies the accuracy of positive prediction made by the model. |
| 3 | Recall | $\frac{TP}{TP+FN}$ | Indicates the model’s ability to capture all positive instances, without missing any. |
| 4 | F1-score | $\frac{2(Precision*Recall)}{Precision+Recall}$ | It is useful metric for models with imbalanced classes |
| 5 | AUC-ROC | $\sum_{i=1}^{n-1} \frac{\left( {FP}_{i}- {FP}_{i-1} \right). \left( {TP}_{i}+ {TP}_{i-1} \right)}{2}$ | Quantifies the model’s ability to distinguish between the positive and negative classes across different threshold values. |

Where TP, TN FP, and FN stand for True Positive, True Negative, False Positive and False Negative, respectively.
