# Supplementary material for: Using transformers and Bi-LSTM with sentence embeddings for prediction of openness human personality trait
Source: PeerJ Comput Sci. 2025 May 22;11:e2781. doi: 10.7717/peerj-cs.2781 (PMC12190728; doi:10.7717/peerj-cs.2781)
Supplement: Supplemental Information 5 [file peerj-cs-11-2781-s005.docx]

**Limitations/validity**

One limitation of this study is that it focuses exclusively on the prediction of Openness personality trait. While Openness is a critical dimension of personality trait, there are other equally important traits that also significantly contribute to the overall understanding of individual differences in behavior. Moving forward, we will explore this research by adding more traits using LLM techniques, thereby enhancing the applicability of and robustness of our findings across the full spectrum of personality dimensions.
